# Water, sanitation, and hygiene in soil-transmitted helminth control: A scoping review of evidence gaps across transmission pathways

**DOI:** 10.1371/journal.pgph.0006196

**Published:** 2026-10-22

**Authors:** Yael Velleman, Anne Straily, W. Evan Secor, Susana Vaz Nery, Laura Braun

**Affiliations:** 1 Policy and Innovation Team, Unlimit Health, London, United Kingdom; 2 Division of Parasitic Diseases and Malaria, US Centers for Disease Control and Prevention, Atlanta, Georgia, United States of America; 3 The Kirby Institute, Sydney, Australia; 4 Environmental Health Group, London School of Hygiene and Tropical Medicine, London, United Kingdom; PLOS: Public Library of Science, UNITED STATES OF AMERICA

## Abstract

Soil-transmitted helminths (STH) are parasitic intestinal worms infecting humans through contact with, or ingestion of, soil contaminated with eggs shed by infected individuals. STHs include roundworms (*Ascaris lumbricoides, Strongyloides stercoralis),* whipworm (*Trichuris trichiura*) and hookworms (*Ancylostoma duodenale*, *Necator americanus, Ancylostoma ceylanicum*). Their transmission is influenced by water, sanitation and hygiene (WASH) services and practices. Unlike past reviews, this scoping review did not assess effect sizes or strength of associations. Instead, it aimed to: map WASH-related risk factors in relation to STH infection; assess whether these align with plausible transmission pathways and conceptual frameworks; and characterise methodological features of the evidence base, including diagnostic approaches, that affect comparability of findings. We analysed 197 studies, of which 174 were observational and 23 were intervention studies. We found a substantial and growing body of evidence examining the relationship between WASH and other environmental exposures and the prevalence and intensity of STH infection. Nonetheless, important aspects such as food chain pathways, environmental surveillance, under-studied species, and measures of service and infrastructure uptake and use, quality, and community coverage over time, remain under-investigated, hampering drawing practical conclusions for improvement of service delivery. Existing and new research can be enhanced in several areas. Routine investigations like prevalence surveys should apply standard WASH definitions to reduce heterogeneity, and apply a conceptual framework of transmission in study design. Intervention studies should be based on theories of change that map actions disrupting transmission pathways, implementing a comprehensive intervention package and examining factors affecting behaviours or intervention compliance. Evaluation of intervention uptake should also be included. New research should address the evidence gaps identified, and incorporate timelines with realistic likelihood of impact into study planning and proposals, given the environmental persistence of certain STH species and the fact that developing lasting and sustained behaviour change can be slow.

## Introduction

Soil-transmitted helminths (STH) are a group of parasitic intestinal worms that infect humans through contact with, or ingestion of, soil contaminated with eggs shed by infected individuals. The group includes roundworms (*A. lumbricoides* and *Strongyloides stercoralis*), whipworm (*T. trichiura*), and hookworms (*Ancylostoma duodenale*, *Necator americanus* and *Ancylostoma ceylanicum*) [1,2]*.* Transmission occurs when excreta of infected humans enter the environment through open defecation, breakdown or disruption of safe sanitation systems, or use of faecal matter for agricultural purposes [3]. In the case of *A. lumbricoides* and *T. trichiura*, infection takes place when humans ingest infective eggs, usually through hand contact with contaminated soil or consumption of unwashed food crops or potentially contaminated water [3]. Hookworms and *S. stercoralis* are transmitted through skin contact with infective larvae in contaminated soil, usually when walking barefoot, with some infection through ingestion possible for hookworm (particularly *A. duodenale* and *A. ceylanicum*) [3]. *S. stercoralis* is also capable of autoinfection. Except for autoinfecting *S. stercoralis,* worm eggs or larvae must mature in soil to become infective. STH infections are common globally and are estimated by the World Health Organization (WHO) to infect around 1.5 billion people worldwide [4].

The transmission pathways of STH infections are heavily influenced by the availability, accessibility, affordability, acceptability and quality of water supply, sanitation and hygiene (WASH) services, and practices. This relationship has been previously explored through several systematic reviews and meta-analyses [5–7]. Strunz et al (2014) conducted a systematic review and meta-analysis of 94 studies to summarise the relationship between WASH access and STH infection [5]. The authors reported a reduction in odds of infection of at least 33% with individual-level WASH practices or access that included not only sanitation but also water and hygiene access. With most studies having an observational design, the overall quality of evidence was deemed low, and lack of standardised WASH definitions limited generalisation across studies.

In a subsequent systematic review and meta-analysis of 65 studies focusing on the role of sanitation alone in disease transmission, Freeman et al (2017) found that sanitation was associated with lower odds of infection with all four included STH species, although heterogeneity was high [6]. The authors found evidence of a protective association between any sanitation (presence or use) and STH infection, compared to no sanitation access. However, there were no significant differences in prevalence or infection between intervention and control arms reported and there was no evidence for associations between improved sanitation and infection, compared to unimproved sanitation. Using the Grading of Recommendations, Assessment, Development, and Evaluation (GRADE) approach, the quality of the evidence was considered ‘very low’ or ‘low’.

A later review by Vaz Nery et al (2019) summarised the dearth of trial evidence on WASH and STH infection [7]. Existing evidence showed mixed results, which attribute to inappropriate or overly complex WASH interventions, resulting in limited uptake or sustained use, as well as concurrent deworming. Additionally, as most study settings already have *some* service access, rather than *none*, a threshold level of coverage and use that will result in interrupting transmission pathways sufficiently to reduce infection is difficult to establish and will vary significantly depending on setting-specific social and environmental factors [7].

Finally, a systematic review and meta-analysis of interventions to improve WASH for preventing STH was conducted by Garn et al (2022) [8]. Based on findings from 32 studies (16 randomised control studies (RCTs) and 16 non-RCTs), the authors concluded that the WASH interventions provided a small protective effect against STH infections, as well as for other diseases transmitted through the faecal-oral route. Given the relatively small protective effect, the authors highlighted the need for rigorous and targeted implementation research and process evaluations to increase the benefit of future WASH interventions.

### Background to the review

Targeted preventive chemotherapy (PC) with benzimidazoles (except for *S. stercoralis*, which is treated with ivermectin) has been the focus of STH control for the past two decades [9]. More recently, as part of the wider drive to end neglected tropical diseases (NTDs) laid out in the NTD road map 2021–2030 [4], there has been a shift towards elimination of STH as a public health problem (EPHP). The WHO defines EPHP as <2% proportion of STH infections of moderate and heavy intensity due to *A. lumbricoides*, *T. trichiura*, *N. americanus* and *A. duodenale* [4]*.* However, reaching EPHP targets is hindered by the ubiquity of STH infections, the resilience of parasites in the environment, the multiple and complex transmission pathways that require many context-specific interventions, continued challenges in access to safely managed WASH services, and heavy reliance on PC programmes mainly targeting school age children and using donated drugs [4].The WHO roadmap also includes preschool age children and women of reproductive age as risk groups.

As part of efforts to meet the ambitious targets set out in the 2021–2030 NTD road map, in 2021 the WHO Department for the Control of Neglected Tropical Disease set up the Technical Advisory Group (TAG) on Soil Transmitted Helminthiasis and Schistosomiasis Control and Elimination [10]. The TAG was designed to act as an advisory body to WHO, providing technical advice on support to countries on programme implementation and policy development. Recognising the importance of WASH for the prevention and elimination of schistosomiasis (SCH) and STH, a dedicated WASH sub-group was established to: a) synthesise and disseminate programme-relevant information on WASH for the control and elimination of SCH and STH; b) support WHO in the development of policy positions on WASH for SCH/STH control; and c) identify and recommend areas for future research. The sub-group agreed that the link between WASH, associated behaviours, and STH transmission is well established. Rather than undertaking another systematic review of associations, the sub-group agreed that a scoping review [11] would be most appropriated.

This scoping review aims to (1) map which WASH-related risk factors have been examined in relation to STH infection across observational and intervention study designs; (2) to assess the extent to which these risk factors, and how they were measured, align with biologically plausible, species-specific transmission pathways and existing conceptual frameworks; and (3) to describe methodological characteristics of the evidence base, including study designs, exposure definitions and diagnostic approaches, that affect the comparability and interpretability of findings across studies. A related review on WASH and schistosomiasis has been published [12].

## Methods

### Protocol and registration

The review protocol was registered on the Open Science Framework registries website on 10 October 2022. Registration DOI https://doi.org/10.17605/OSF.IO/BTNHE.

### Search strategy

All information sources are described in the PRISMA flow diagram (Fig 1). Five electronic databases were searched during August-September of 2022 and updated in May 2025: Medline, Embase, Cochrane, Global Health, and Scopus. Additionally, a call for papers was shared in March 2022 through various networks and channels, including the NTD NGO Network membership list, the Sustainable Sanitation Alliance (SuSanA) Forum, the WHO PHE newsletter mail-out, and through social media (Twitter (X) and WhatsApp). The list of included studies from the previous systematic review on WASH and STH [5] was then added to the reviewed studies; one publication (Chongsuvivatwong, 1996 [13]) was treated as two studies in the Strunz et al [5] review and this distinction was retained in our analysis. Studies included by Strunz et al [5] were retrieved and reviewed separately, and the inclusion criteria were applied, resulting in further exclusions.

**Fig 1:**
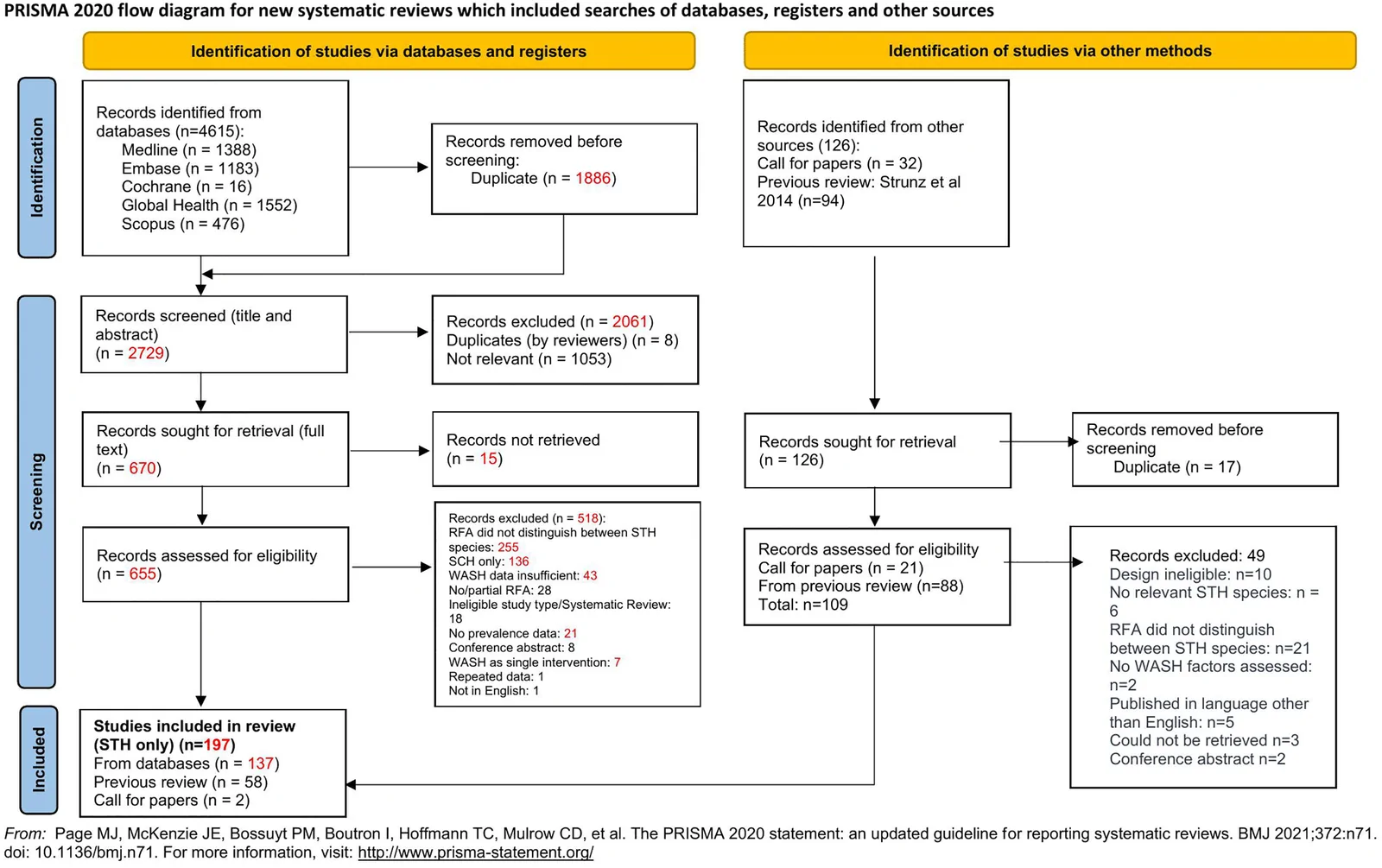
PRISMA flow diagram.

The search strategy was informed by a conceptual framework (Fig 2) setting out all likely transmission pathways and their presumed relationship with water supply, sanitation and behaviours (hygiene as well as shoe wearing). The strategy focused on three primary categories: 1 - STHs, 2 - WASH, and 3 - infection mechanism. The full list of search terms is included in Text A in S1 Appendix.

**Fig 2:**
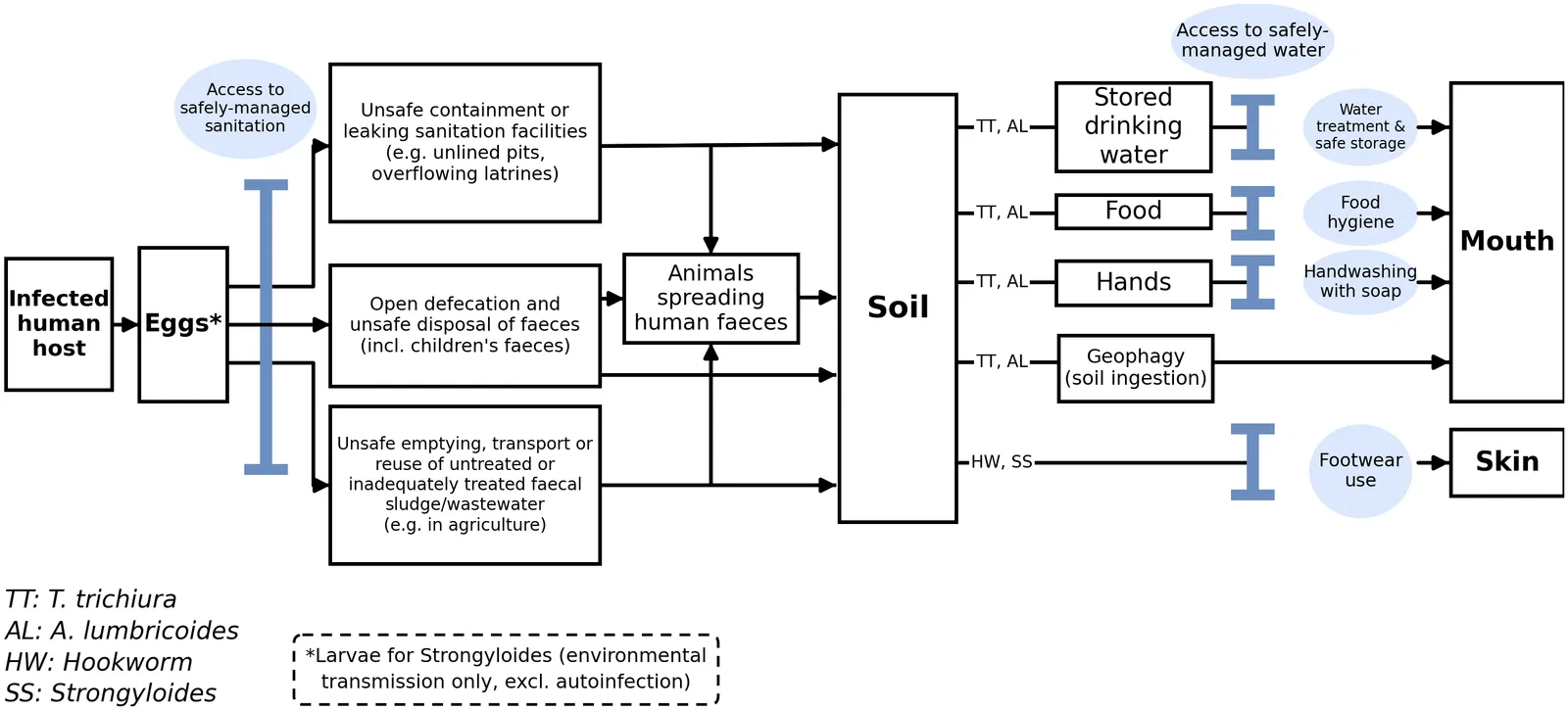
STH transmission pathways. The diagram sets out the predominant transmission pathways of STH species from one host to another from left to right. Arrows show the pathway of parasite eggs from the host to the environment (soil), and infective stages of the parasite into the new host. Vertical blue lines represent WASH services and behaviours acting as potential barriers to transmission. Autoinfection in the case of *S. stercoralis* is not included in this diagram. *T. trichiura* (TT), *A. lumbricoides* (AL), hookworm (HW), *S. stercoralis* (SS).

### Eligibility criteria

Eligible study designs included intervention studies (Randomised Controlled Trial, Quasi-randomised Controlled Trial, Controlled Before and After Study, Contemporaneous data collection, Comparable control sites with at least 2 x intervention and 2 x control clusters, Interrupted Time Series with at least 3 time points before and 3 after the intervention, and a clearly defined intervention point) and observational studies (all designs except ecological studies). The literature search focused on studies published since 29 October 2013, inclusive, to capture studies published since the last comprehensive systematic review and meta-analysis [5]. Only studies published in English and having both exposure (e.g., quantified access to water or sanitation or hygiene) and outcome measures (prevalence/intensity of infection) for STH infections were considered. Studies reporting only pooled STH infection, rather than species-specific outcomes, were excluded. We only included studies that examined specific WASH activities for individual parasite species. For example, studies that reported results for a combined WASH package or presented infection data only as “any STH”, were excluded. Only peer-reviewed articles were included.

### Data selection, data extraction and analysis

Search results were de-duplicated in Endnote and uploaded to Rayyan [14]. Titles and abstracts were double screened, split among four reviewers (YV, ES, LB, AS). Any disagreements were discussed by the reviewers, with any unresolved disagreements discussed with the other reviewers. Full texts meeting the inclusion criteria were retrieved and double screened by two reviewers in Excel (Microsoft, Redmond, WA, USA). Given that SCH and STH are often studied simultaneously, the search, abstract and full text review were conducted jointly. The review was split between the two diseases at the data extraction phase.

Data of included full text articles were extracted using a pre-specified data extraction sheet by two reviewers (see S1 Data). Extracted data included study metadata (e.g., authors, year of publication, title, country, study design, diagnostic methods, setting (urban/rural), participant numbers and ages, and study objectives) and specific WASH variables. WASH variables of interest included water, sanitation, animal presence, food and hand hygiene, and shoe wearing (see Table A in S1 Appendix for full list). WASH variables were extracted even if they did not appear in the conceptual framework. All relevant variables were extracted, even where the definitions used by studies did not match the anticipated variables. For instance, all information on water sources or sanitation was extracted even if definitions of ‘improved/unimproved’ were not applied. As this was a scoping review, no meta-analyses of extracted data were undertaken. Extracted data were summarised according to the conceptual framework to identify strength and availability of evidence and identify gaps in research.

## Results

The search identified 4,741 records: 4,615 from databases, 32 from the call for papers, and 94 from the previous review (Fig 1). After deduplication, 2,729 titles and abstracts were screened. A total of 762 full text articles were screened (655 identified through the database search, 21 through the call for papers, and 88 from the previous review), resulting in 197 included studies.

Many studies (n = 567) were excluded at full text review stage. The most common reasons included analysis not distinguishing between STH species; insufficient or absent WASH data; ineligible study type or systematic/literature review; no disease prevalence specified; or combined WASH activities described as a single intervention (Fig 1). The most frequent cause of study exclusion (276 studies) was failure to distinguish between STH species in the analysis. Given the distinct transmission pathways – faecal-oral for *A. lumbricoides* and *T. trichiuris* compared with skin penetration for hookworm species and *S. stercoralis* – it would not have been possible to separate the exposures from the infection outcome. Notably, in all cases, this ‘lumping’ of species occurred during risk factor analysis despite those same studies assessing species prevalence separately. Studies focussed on SCH only were excluded from this review but included in the parallel review on WASH and SCH.

A total of 197 studies were included in this review. Of these, 131 (66.5%) were conducted in rural settings, 24 (12.1%) in urban areas, 24 (12.1%) in both, and 18 (9.1%) did not indicate the setting. Participant numbers ranged between 95 and 33,363 per study. Over half (108/197, 54.8%) of studies reported on multiple STH species. Of the remaining 89 studies, 44 reported on hookworm *spp.* only, 28 on *A. lumbricoides* only, 15 on *S. stercoralis only,* and two on *T. trichiura* only.

### Observational studies

Overall, most included studies (88.3%, 174/197) were observational (see Table B in S1 Appendix for the complete list). Of these, most (163/174, 93.6%) had a cross-sectional study design to measure prevalence and assess risk factors, including at least one WASH variable or behavioural factor. Observational studies were conducted in Africa (81/174, 46.5%), Asia and the Pacific (68/174, 39%), and the Americas and Caribbean (25/174, 14.3%). Most (49.5%, n = 86) were community-based, followed by school-based studies (n = 52, 29.8%), with only one study including both school and community settings. The remainder were health facility-based or focused on specific occupational or other sub-groups (e.g., plantation workers, street beggars).

Most observational studies (114/174, 65.5%) only reported prevalence of infection, while two (1.1%) reported infection intensity only (eggs per gram of stool), and 58 (33.3%) reported both. Most risk factor analyses were based on infection prevalence.

Fig 3 summarises the number of observational studies that reported WASH variables by species, defined using the conceptual framework (Fig 2) as part of the risk factor analysis (Table C in S1 Appendix). Hookworm and *A. lumbricoides* were most frequently studied, followed by *T. trichiura* and finally *S. stercoralis*. Most studies examined multiple species simultaneously. For all species, access to improved sanitation was the most frequently analysed variable (85% of studies), followed by improved water access (50%), and practice of handwashing (40.8%). In some areas, this relationship is not clear: for instance, two hookworm species (*A. duodenale* and *A. ceylanicum*) can be transmitted through ingestion while others cannot. Handwashing likewise plays a part in interrupting transmission when done before eating, while handwashing after toilet use is less directly linked to transmission. Many studies that included information on handwashing practice referred specifically to handwashing after toilet use. This is highly relevant for the prevention of many faecal-oral infections, but is arguably less relevant to STH, as it ignores the need for eggs to mature in soil before becoming infective. However, handwashing after toilet use may be indicative of broader hygiene practices. Surprisingly, only 46.2% of studies that included hookworm outcomes examined shoe wearing practices, despite the plausible link between walking barefoot and the risk of hookworm infection.

**Fig 3:**
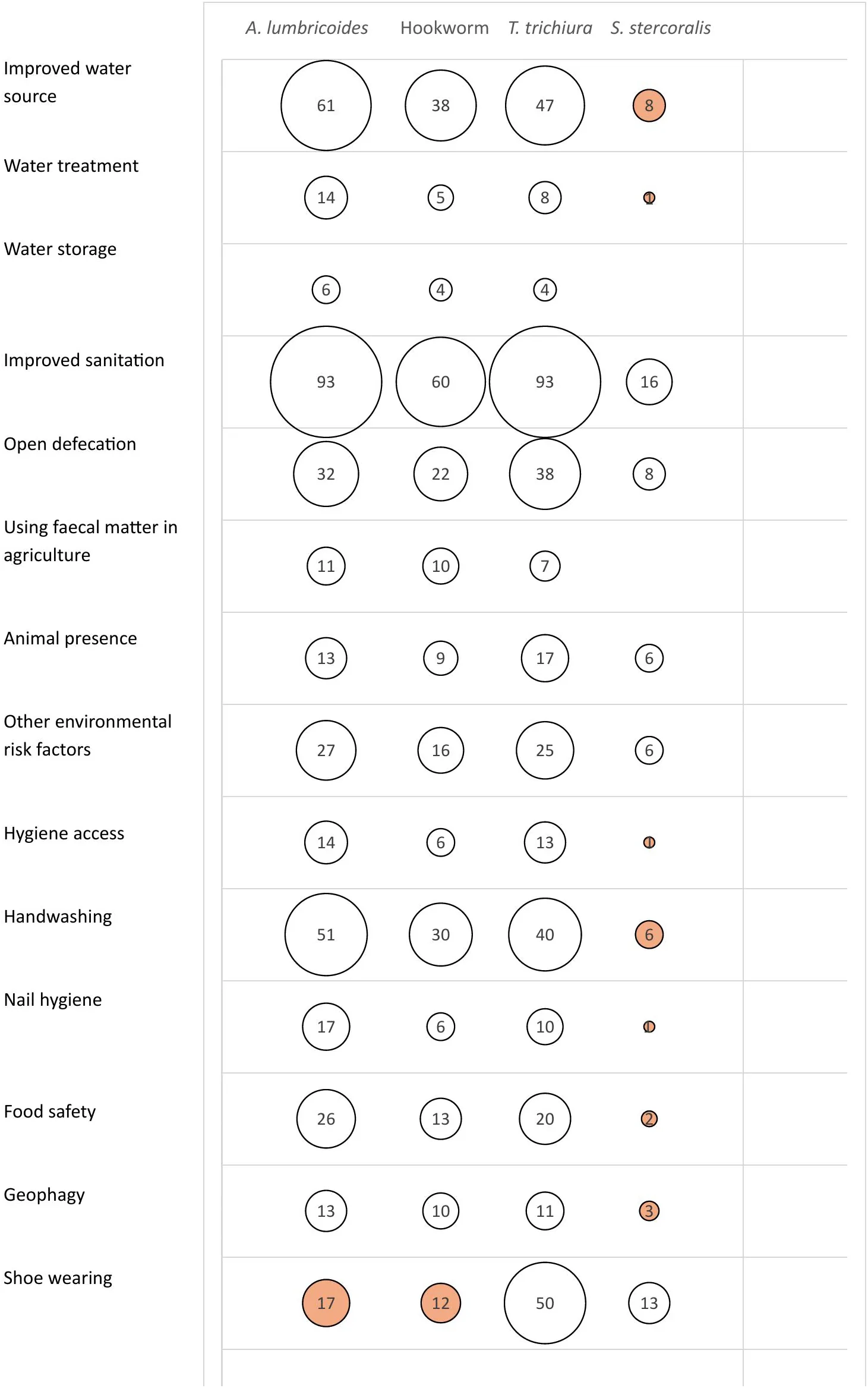
Number of observational studies reporting on WASH relationship with infection and/or intensity for each WASH parameter, by species. The size of the circles denotes the number of studies, and circles highlighted in red denote occasions in which WASH variables were examined in the study despite not being part of the transmission pathway (for instance, shoe wearing and *A. lumbricoides/ T. trichiura*).

### Availability/strength of evidence

Fig 4 plots the relative availability of observational studies for each component of the conceptual framework of transmission regardless of the STH species studied. Access to water supply, toilets and handwashing practices were the most studied WASH aspects (grouped under “sanitation” in Fig 4). This possibly represents not only the obviousness of these categories as subjects of inquiry, but also the ease of including such aspects in questionnaires – a method applied in most cross-sectional studies to undertake risk factor analyses – rather than data collection by observation of behaviours or facilities. For example, fewer than half of the studies that included information on access to toilets also addressed open defecation practices (55/148) and only 21/174 (12%) of studies examined access to handwashing facilities. Water storage was the least studied category (6/174, 3.4%) despite the importance of this pathway for transmission of *A. lumbricoides*, *T. trichiura*, and some hookworm species. Although 41/174 observational studies evaluated one or more environmental factors, when examined individually, these factors are considerably under-studied, including type and material of flooring or walls in the home (13/174), methods of household waste disposal or presence of waste near home (9/174), proximity to/use of water bodies/rivers (6/174), habit of playing with soil (5/174), irrigation (5/174), contact with wastewater (3/174), visible faeces (2/174), latrine cleanliness (2/174), drainage (2/174), contact with ground while working (2/174), soil type (1/174), ratio of children per toilet in school (1/74), and precipitation (1/174). Only 33.9% of studies included information on open defecation. Several studies implied the potential of open defecation practice, e.g., by referring to inconsistent use of toilets (always, sometimes, never), inconsistent use of designated areas for defecation, or absence of household or school latrines. However, we did not assume that these studies measured open defecation unless this was explicitly mentioned. These gaps and inconsistencies demonstrate the failure of most observational studies to apply a conceptual framework of disease transmission pathways.

**Fig 4:**
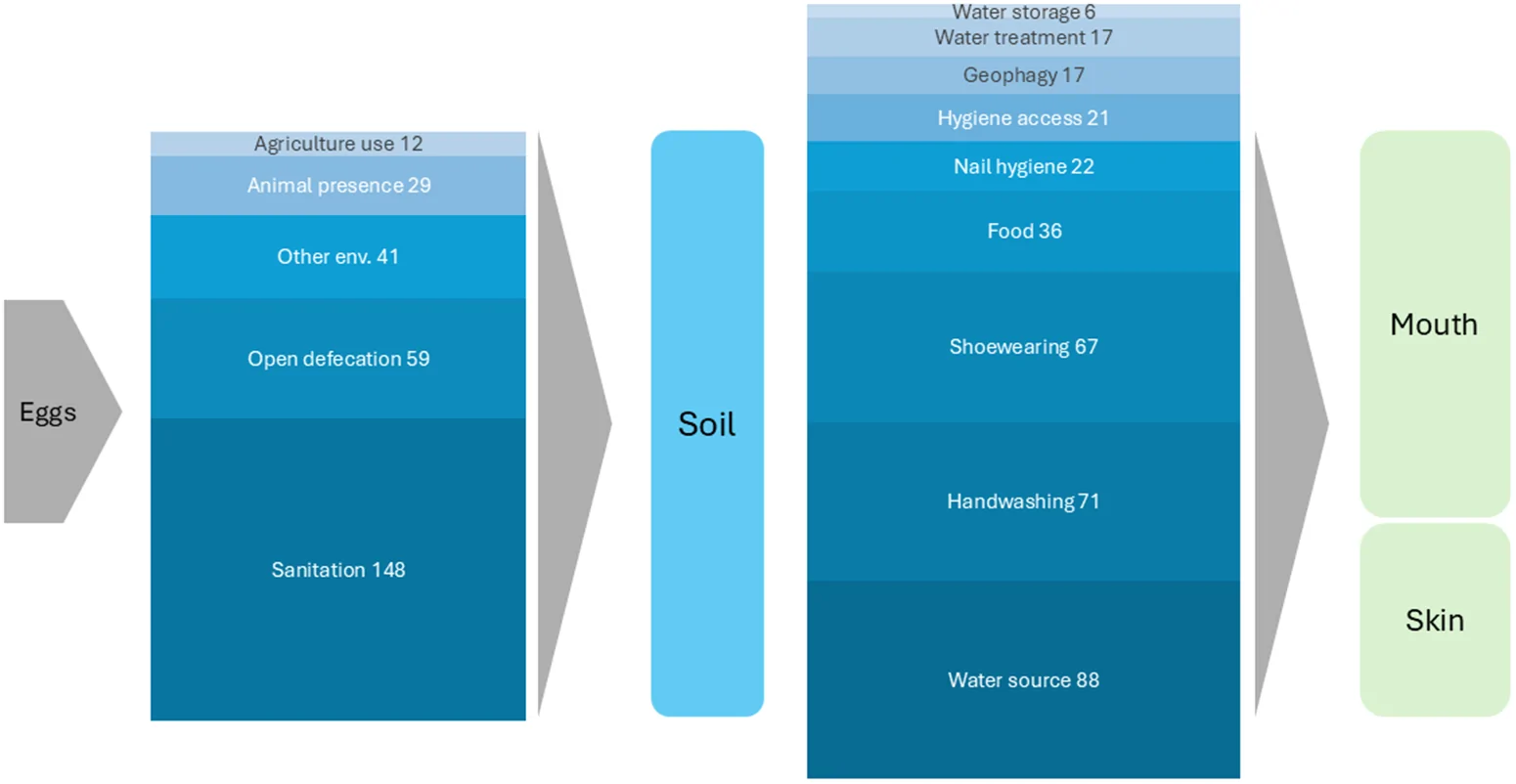
Number of studies reporting on each of the WASH-related transmission pathways.

There was significant heterogeneity in how factors were defined across observational studies. The few studies (29/174) examining animal presence looked at a wide variety of livestock and pets, estimating physical contact and/or presence within the home or compound. There was also significant heterogeneity in how shoe wearing behaviours were assessed; most studies used some combination of the categories “always, often, sometimes, never”, but it was not consistent nor were the categories defined. Food safety aspects examined were also varied and included activities such as eating uncooked, unwashed or unpeeled fruit and vegetables, handwashing before eating/cooking, eating street food, utensil use, and consumption of raw meat or fish (the latter is not included in any STH transmission pathway). This heterogeneity in how factors were defined precluded a comprehensive analysis of risk factors across studies and limits interpretation and generalisability.

The included studies overwhelmingly examined associations between individual or household level exposure (WASH conditions and associated behaviours), and individual outcomes (STH species infection). Even studies that looked at open defecation practices mostly did not note or analyse the proportion of the population in each setting undertaking such practices and, by implication, affecting the level of risk of transmission in that setting. Some exceptions were noted; Barreto et al. found that increased neighbourhood sewage system coverage in Brazil reduced *A. lumbricoides* and *T. trichiura* prevalence, concluding that such coverage prevented transmission mainly in the public, rather than the household, domain [15]. In Kenya, Oswald et al. noted that village sanitation coverage over 80% was associated with reduced prevalence of *T. trichiura* infection [16]. Riess et al. found a significant association between latrine coverage in household surroundings and hookworm infection overall [17]. Conversely, Oswald et al. reported no evidence of a protective effect of increased community sanitation usage in Amhara, Ethiopia, noting the complex relationship between infection and community sanitation usage, and lack of inclusion of other potentially important factors in the study [18]. Campbell et al. found little to no effect of village-level WASH access on hookworm and *A. lumbricoides* infection intensity in Timor-Leste, although noting that WASH access was generally poor in the study communities, which likely limited their ability to find any associations [19].

### Intervention studies

Twenty-three intervention studies met the inclusion criteria for this review (Table D in S1 Appendix). Most (n = 15) employed a cluster or community level randomised control trial design. Ten studies examined intervention impact on three STH species (*A. lumbricoides, T. trichiura* and hookworms), with four studying hookworm only, three studying both *T. trichiura* and *A. lumbricoides*, and two focused solely on *A. lumbricoides*. Two studies reported A. *lumbricoides* and hookworm outcomes, one hookworm and *S. stercoralis*, and one *T. trichiura* and hookworm. Only one study included *S. stercoralis* outcomes. Among the intervention studies, most (19/23) reported both prevalence and intensity, while four measured prevalence only.

Seven studies (30.4%) implemented a comprehensive package of WASH interventions [20–26] while the remaining studies [27–42] explored one or two WASH aspects (Table 1). No study focused solely on water and only seven studies (30.4%) included a water intervention. Of these, three focused solely on water quality (treatment and storage), and four on access (provision of water sources). Sanitation interventions were included in 17/23 intervention studies, ranging from latrine construction and upgrading (n = 10), community-led total sanitation (n = 2), addition of child potties alongside latrine improvements (n = 2), Participatory Hygiene and Sanitation Transformation (PHAST) (n = 1), and subsidies and sanitation marketing (n = 2). Twelve (14/23) studies included hygiene interventions, of which only two included provision of handwashing facilities (the WASH-B trials in Bangladesh [22] and Kenya [24]), one included demonstrations of constructing handwashing facilities [37], one included provision of towels and training in alcogel production [38] and one included provision of bathing facilities [20]. One study [27] tested only the distribution of shoes and impact on hookworm prevalence, with no other WASH interventions. Ten studies included broader health education interventions, while two (the WASH-B trials in Kenya [24] and Bangladesh [22]) also included a nutrition intervention of breastfeeding promotion and nutrient supplements.

**Table 1:** Summary of intervention study results categorised by intervention components. An expanded summary is included in Table D in S1 Appendix.

Intervention package	Intervention type	Study	Setting	Duration of intervention/ follow up	Deworming treatment	Reduction in prevalence, infection intensity or re-infection
**Single intervention**	Shoe wearing	Bird et al [27]	School	One off distribution/ 6 months	At baseline	☒
	Hygiene	Al Delaimy et al [41]	School	6 months/ 6 months	Prior to intervention	□
		Bieri et al [28]	School	One school year/ end of school year (10 months)	At baseline	☑
		Gyorkos et al [29]	School	4 months of intervention and immediate follow up	At baseline	□
		Makata, et al [30]	School	12 months/ 12 months after baseline	Prior to baseline survey and again at baseline for remaining infections.	☒
		Mahmud, et al [42]	Community	6 months/ at end of 6-month intervention period	Before screening to exclude children diagnosed positive	☑
		Nachaiwieng, R. et al [38]	School and community	6 months/ at 3 months and 6 months following implementation	Prior to intervention and after follow-up	☑
	Sanitation	Clasen, et al [31]	Community	12 months/ 18 months	At baseline	☒
		Hurlimann, et al [32]	Community	7 months/ 12 months	Annual treatment shortly after baseline survey, second round at eight months	☒
		Knee, et al [33]	Community (urban compounds)	13 months/ 24 months	At baseline	□
		Vonghachack Y et al [39]	Community	12 months	Before and after intervention	□
**Two interventions**	Sanitation + hygiene	Clarke, et al [34]	Schools and community	6 months/ 6 months	At baseline and six months before follow-up (in one school before intervention completion).	□
		Patil, et al [35]	Community	21 months/ at end of intervention	None specified	☒
	*Sanitation + health education*	Sayasone, S et al [40]	Community	13 months/ 12 months after intervention	Before and after intervention, then after follow up study	☑
		Steinmann, et al [36]	Community	36 months/ 24 months post implementation	MDA at follow up	☑
**Three interventions**	Sanitation + health promotion + handwashing facilities	Dumba, et al [37]	Community	Unspecified	Every three months (unclear over what period).	☑
**Comprehensive WASH:** water supply, sanitation and hygiene interventions		Reese, et al [20]	Community	36 months/ 5 years post implementation	None specified	☑
		Tadesse, G et al [26]	School	22 months/ one year into delivery and at the end of the study	At the start of the study and twice more annually	☒
		Vaz Nery, et al [21]	Community	11 months/ 4 times over 24 months	Every six months over two years (five rounds).	☒
**Comprehensive WASH + :** water supply, sanitation and hygiene interventions implemented alongside other health interventions (e.g., nutrition, comprehensive health education)		Ercumen, et al [22]	Community	25 months/ 30 months	2/3 of children were dewormed within 6 months prior to assessment (2 months on average).	☑
		Freeman, et al [23]	School	18 months/ follow-up 1 at 11 months, follow-up 2 at 21 months	At baseline and follow up 1 and 2 (not during the intervention).	□
		Pickering, et al [24]	Community	24 months/ 27 months	National school-based MDA undertaken during study. 43% of participants dewormed in the past 6 months	☑
		Zeng, et al [25]	Community	36 months/ 5 years	Annual MDA over the three years to the entire population except pregnant women and children under 3	☑

☒ No statistically significant effect of the intervention on STH outcome(s).

**□** Statistically significant effect on STH outcome(s) in some but not all sub-groups or intervention arms.

☑ Intervention resulted in statistically significant effect on STH outcomes.

Table 1 provides a breakdown of the included intervention studies, including the type of intervention applied, the duration, and whether and when deworming treatments were given. Of the 23 intervention studies included in this review, ten (43.4%) achieved a reduction in STH prevalence and/or intensity of infection in the intervention arm [20,22,24,25,28,36–38,40,42]. Among these, six included a comprehensive WASH intervention package that addressed multiple aspects of the conceptual framework, providing improved sanitation alongside improvements in drinking water or handwashing facilities and health education or health promotion activities to increase behaviour change. At the same time, three studies using a comprehensive intervention package reported no or inconsistent impact [21,23,26]. Reasons provided by authors included small sample sizes, potential masking of differences between intervention and control groups by deworming and WASH improvements, short follow-up, initial low prevalence levels, insufficient change to infection risk (i.e., continued inadequate WASH practices), and inability of the intervention to cover non-school exposures. An expanded summary of intervention study design and results is provided in the supplementary materials (Table D in S1 Appendix).

Diagnostic approach is a key methodological characteristic affecting the comparability of infection outcomes across studies. Many studies (n = 48), especially those that looked at more than one STH species, utilised multiple diagnostic techniques performed on the same stool sample (Table 2). In some cases, it was not specified which test was used for which species, even where the testing modality might not be ideal for a particular parasite species (e.g., Kato Katz or direct smear for *S. stercoralis*). The Kato Katz method was by far the most frequently employed diagnostic test among included studies: 43.9%, 78.8%, and 71.2% of studies used Kato Katz to diagnose *A. lumbricoides*, *T. trichiura*, and hookworm, respectively. Most studies analysed two slides from a single stool sample. Other commonly used methods include Formalin-Ether Sedimentation/ Concentration methods and direct smear. Of the studies that included *S. stercoralis*, 19% also reported using Kato Katz although it is not an appropriate method by which to identify this infection. Molecular methods were used in 11% of studies.

**Table 2:** Diagnostics for determining the prevalence of STH used in included studies.

	*A. lumbricoides*	*T. trichiura*	Hookworm	*S. stercoralis*
**Diagnostic test**	**% (n)**	**% (n)**	**% (n)**	**% (n)**
Kato Katz	43.9% (80)	78.8% (56)	71.2% (77)	19% (4)
Formalin-Ether Sedimentation/ Concentration	14.2% (26)	23.9% (17)	22.2% (24)	33.3% (7)
Direct smear/ wet mount microscopy	8.7% (16)	14% (10)	16.6% (18)	19% (4)
Koga Agar Plate Culture	1.6% (3)	4.2% (3)	3.7% (4)	71.4% (15)
Baermann	1.6% (3)	4.2% (3)	3.7% (4)	52.3% (11)
qPCR/PCR/RT-PCR	3.2% (6)	1.4% (1)	7.4% (8)	19% (4)
Harada-Mori filter paper culture	2.7% (5)	9.8% (7)	4.6% (5)	
Spontaneous/ centrifugal flotation, Sugar/Salt flotation technique: Sheather/ Willis/ Faust	2.1% (4)	5.6% (4)	2.7% (3)	4.5% (1)
FLOTAC/ Mini-FLOTAC	1.6% (3)	1.4% (1)	2.7% (3)	
Spontaneous sedimentation/ spontaneous tube sedimentation technique/ simple sedimentation/ Telemann	1.6% (3)	4.2% (3)	0.9% (1)	9.5% (2)
McMaster	1.0% (2)	2.8% (2)	2.7% (3)	
Ritchie Technique	0.5% (1)	1.4% (1)	2.7% (3)	
NIE-ELISA				19% (4)
Sedimentation and flotation (specific method not mentioned)	0.5% (1)		1.8% (2)	4.5% (1)
ethyl-acetate sedimentation	0.5% (1)	1.4% (1)	1.8% (2)	
Rugai, Mattos and Brisola Technique	0.5% (1)	1.4% (1)	0.9% (1)	
Fecal Parasite Concentrator/ Evergreen	1.0% (2)	1.4% (1)	0.9% (1)	

## Discussion

This scoping review mapped WASH-related risk factors examined in relation to STH infection, assessed their alignment with plausible transmission pathways and conceptual frameworks, and characterised the methodological features shaping the evidence base’s comparability and interpretability. We found a substantial and growing body of observational and intervention research, but one that draws on a narrow and inconsistently applied set of risk factors given the range of plausible transmission pathways set out in our conceptual framework (Fig 2). Few studies were explicitly grounded in a conceptual framework of transmission, and this likely explains two things at once: biologically relevant exposures being left out (e.g., food chain contamination, environmental persistence of eggs) and exposures of limited relevance to a given species being included instead. The evidence base also had considerable heterogeneity in how exposures were defined, heavy reliance on self-reported WASH access and practices, and variable diagnostic approaches. These issues make it difficult to generalise risk factor findings, limiting the conclusions that can be drawn for research and programme design in support of STH control and elimination objectives, and informing our considerations for future research (Table 3).

**Table 3:** Priorities for future research.

**Addressing Key Transmission Gaps**	**Food chain contamination.** While some studies examined food safety practices such as handwashing before cooking or eating, or washing of raw fruit or vegetables, none of the included studies examined levels of contamination of crops in fields or in markets, nor the impact of food handler infection or practices on transmission.
	**Environmental presence and persistence of parasite eggs.** Some studies sampled soil for parasite eggs, but none examined the effect of WASH conditions on egg presence or levels of environmental contamination. Studies incorporating environmental surveillance are needed to identify the type, quality, and coverage of infrastructure, particularly sanitation, required to reduce overall prevalence and morbidity. Such studies must consider egg resilience and apply appropriate timescales, given the environmental persistence of species such as *A. lumbricoides* and *T. trichiura*.
	**Quality, uptake and maintenance of services.** The influence of uptake and maintenance practices, associated barriers, and changes in access and use over time remains relatively under-studied compared with simple binary measures of access. Future studies should measure community uptake of WASH interventions and identify barriers (e.g., poor construction or maintenance, disruption of social customs, privacy concerns, location) that reduce use.
	**Access thresholds and community coverage.** Evaluation of access to WASH interventions is needed to elucidate the relationship between different levels of community coverage and reductions in prevalence. Access must be meaningful: moderate increases that leave a substantial proportion of the population practising open defecation are unlikely to sufficiently reduce exposure. Similarly, universal coverage with basic but poorly managed latrines may not result in significant reductions in infection. Longitudinal measures of uptake and use should therefore be incorporated.
	**Multiple exposure settings.** Studies should develop a more holistic picture of communities’ overall WASH environments, including all locations where people spend time and may defecate or be exposed (e.g., home, school, and work).
	**Strongyloidiasis.** Strongyloidiasis remains under-studied compared with other STH species. Researchers targeting *Strongyloides stercoralis* should explicitly account for its autoinfective cycle, which may limit the detection of WASH or behaviour change effects and was not incorporated into the conceptual framework used in this review.
**Transmission-Informed Study Design**	**Standardised definitions:** Significant heterogeneity in terminology and measurement limits comparability across studies. Applying standard definitions of infrastructure and service levels, such as those recommended by the WHO/UNICEF Joint Monitoring Programme [74] would reduce heterogeneity.
	**Study design:** Future studies should apply a species-specific conceptual framework of transmission to guide study design and variable selection, distinguishing clearly between helminth species in risk factor analyses. This approach can help prioritise epidemiologically meaningful variables rather than those that are easier to measure but less relevant to specific transmission pathways.
**Designing More Comprehensive Intervention Studies**	**Intervention studies:** Intervention studies must be sufficiently comprehensive to meaningfully interrupt relevant transmission pathways, as partial or narrowly scoped interventions risk generating inconclusive findings [75]. Future intervention research should:
	1. Apply comprehensive intervention packages aligned with species-specific transmission pathways, ensuring that studies are grounded in a clearly articulated theory of change that explains the causal pathways linking intervention components to expected outcomes, and clearly report gaps and limitations.
	2. Incorporate realistic intervention and follow-up periods that account for environmental persistence and species biology.
	3. Include environmental indicators (e.g., STH egg presence, faecal contamination) within a conceptual framework, alongside epidemiological outcomes, to evaluate intermediate steps along the causal pathway from intervention to infection reduction, allowing explicit testing of assumptions about transmission interruption and environmental persistence.
	4. Rely as much as possible on observed rather than reported data to reduce bias
	5. Assess intervention delivery (fidelity), uptake, and trends in coverage, use, and functionality
	6. Current WHO guidelines [9] advise stopping PC programs once targets are reached and highlight the important role of WASH in sustaining gains achieved by PC. However, uncertainty remains regarding which WASH interventions, at what levels of coverage and functionality, and in which transmission settings, are required to prevent resurgence following PC cessation. While mathematical modelling suggests that preexisting improved WASH conditions can prevent rebound of infections [76], empirical evidence remains limited. Experimental studies are therefore needed to determine how improvements to WASH services influence transmission dynamics.

A key methodological gap is the limited use of conceptual frameworks, which likely contributed to the substantial evidence gaps observed across included studies. Conceptual frameworks play an important role in describing researchers’ understanding of the subject of investigation and associated factors and variables – in this case, the relationship between environmental and behavioural exposures, and disease outcomes – and in defining the study hypothesis [43,44]. The observational studies largely did not employ a conceptual framework of transmission pathways to guide data collection and analysis, resulting in the exclusion of potentially important ones such as the use of human excreta or wastewater for fertiliser application, irrigation, or aquaculture feed. Additionally, this led to the inclusion of risk factors of limited relevance. For instance, several studies on *A. lumbricoides* and *T. trichiura* included shoe wearing in the risk factor analysis, while some *S. stercoralis* studies examined handwashing practice. Hookworm studies examined safe water sources (n = 47) almost as frequently as shoe wearing (n = 50), despite the assumed greater relative importance of the latter as an infection risk factor. Notably, among the intervention studies that were successful in achieving a reduction in STH infection, most applied a more comprehensive package of WASH interventions that addressed multiple transmission factors in the conceptual framework.

Many studies did not comprehensively consider related risk factors, and most cross-sectional studies relied on self-reported WASH access and practices rather than observations. For example, studies that reported presence of toilets or latrines often did not assess whether sanitation facilities were operational or if open defecation also occurred. Additionally, many studies focussed on WASH indicators that are simple to enumerate, such as the physical presence of a water source or toilet, instead of assessing WASH service levels. The limited use of conceptual frameworks likely contributed to the pronounced heterogeneity observed in indicators and methods. Handwashing practices, for example, were frequently measured using self-reported behaviour and vague, undefined categories (e.g., “sometimes” or “often”), instead of quantifying handwashing at key moments. The use of questionnaires rather than observations further constrained the application of robust definitions of service quality and use, such as evaluating water safety or the use and maintenance of sanitation facilities.

Safely managed sanitation and safe water sources are necessary barriers to transmission in the conceptual framework (Fig 2). These can be viewed as ‘public goods’ that affect health and wellbeing at the societal level [45]. At the community level, this means that one household’s risk may be impacted by the conditions or practices of surrounding households or the broader community [46,47]. However, studies generally focused on one setting over another – either the household/community, school or occupational, thereby excluding exposures faced by the same study participants in other settings. For instance, studies conducted at the school level often captured information solely on school-level exposures, while household exposures were either not included in these studies – therefore leaving out a significant exposure element – or captured using questionnaires – leading to the above-mentioned issues with self-reported WASH data and practices. Given that children spend a significant amount of time at school, access to improved WASH facilities is crucial. These school facilities can contribute to the overall impact of WASH interventions on reducing STH infections at the community level. However, children are also exposed to STH outside of school. While several studies [48–51] investigated the combined effects of school and household or community-level WASH, particularly those that used data from school-based surveys, the results were varied. It is undoubtedly challenging to isolate the effects of school-based WASH from non-school factors, which demonstrates the necessity of evaluating all WASH conditions that individuals are exposed to. Additionally, the level of WASH coverage or access needed to consistently reduce community-level STH infection is unclear and varies across communities due to environmental and cultural differences. Importantly, increased access to improved sanitation does not necessarily account for behaviours affecting uptake or use of improvements or other transmission pathways and this was generally understudied in the papers included in our review.

Although we did not formally assess risk of bias, the methodological limitations observed across studies suggest substantial variability in study quality, which should be considered when interpreting the findings. These limitations include reliance on self-reported exposures, incomplete reporting of diagnostics, and short follow-up periods. Variability in the choice of diagnostic test used (Table 2) further contributes to difficulties in comparing studies. Tests with low diagnostic sensitivity can lead to underestimation of infection prevalence and misclassification of outcomes, which may bias estimates of associations between WASH exposures and infection. Test performance also varies by transmission setting; sensitivity is typically lower in low-intensity areas, particularly for microscopic methods [52–54]. Species-specific limitations should be acknowledged, for example, the reduced sensitivity of Kato–Katz for detecting hookworm [55,56]. Stool samples from participants were often subjected to multiple diagnostic methods to detect different species, though the specific test-species pairing was not always reported, even when the chosen test was suboptimal (e.g., Kato Katz or direct smear for *S. stercoralis*). Reliance on a single stool sample, which is common practice, limits sensitivity for microscopy-based STH detection [52,57] and is particularly inadequate for detecting *S. stercoralis* [57,58]. Failure to account for these diagnostic limitations can weaken the strength and validity of inferences regarding WASH and STH transmission pathways.

Infection intensity (i.e., egg counts) was often not reported, and when it was analyses almost always focused solely on associations with prevalence. This is a notable limitation as infection intensity is a more sensitive indicator of transmission dynamics within communities [59–62] and is required to determine whether a community has achieved EPHP status under current WHO definitions. However, not all diagnostics (e.g., concentration methods) can measure infection intensity accurately, and intensity measurements have not been standardised across most laboratory methods beyond Kato-Katz [54,63]. These limitations constrain the ability of studies to evaluate the effects of WASH exposures or interventions on the full spectrum of STH infection outcomes and may lead to less discriminating, incomplete or biased assessments of transmission.

A prior systematic review of WASH interventions for reducing STH infection highlighted the importance of environmental surveillance, noting that exposure measurements are rarely included in intervention studies [7]. Consistent with this finding, few studies in our review incorporated environmental surveillance methods. Helminth eggs can be highly resilient in the environment under favourable soil and temperature conditions. Unembryonated *A. lumbricoides* eggs may persist in soil for up to 15 years [64], and remain infective for several years [65,66]; eggs can survive in sludge/biosolids for at least 15 months, and depending on storage temperature, have been reported to remain infective for up to 23–33 months [67,68]. Based on studies of *T. suis, T. trichiura* eggs may remain viable in soil for up to 11 years [69]. In contrast, infective stages of hookworm *spp.* and *S. stercoralis* are less resilient, lasting just several weeks under favourable conditions [70–72]. Because some WASH interventions may not effectively target persistent environmental reservoirs of STH eggs, transmission from the environment may continue unless those pathways in the conceptual framework (Fig 2) are also addressed. Without environmental surveillance, this cannot be adequately evaluated. The long persistence of infective eggs in the environment also means that measurable reductions in STH transmission resulting from improved sanitation may take many years to appear [73]. Given that most trials we reviewed had a relatively short implementation and follow-up periods of around one to two years, the lack of consistent impact across age groups and helminth species is perhaps unsurprising [7].

### Limitations

Studies may have been missed due to language restrictions and the choice of databases. The exclusion of grey literature and pre-prints means that additional relevant evidence may not have been captured. As a scoping review, the primary aim was to map and characterise the available evidence rather than to evaluate the strength or direction of associations. Therefore, we did not conduct a formal quality or risk-of-bias assessment, in line with established scoping review methodology. As a result, the findings include evidence of varying quality without formal differentiation. This review focused on WASH variables and transmission pathways; therefore, broader socio-economic and behavioural aspects were not included in our analysis, even if these can theoretically impact on WASH access or practices.

### Priorities for future research on WASH and STH transmission

The review findings provide helpful insights into future research subjects and methods in support of the role of WASH factors in progress towards control and elimination of STH. Rather than re-evaluating the importance of WASH for achievement of public health goals, we assessed the alignment between current study designs and the evidence needed for programme design and delivery. To strengthen comparability across studies and generate policy-relevant evidence, we propose the following priorities for future research (Table 3).

Although these recommendations may increase study costs, more comprehensive and standardised study designs are likely to generate stronger and more interpretable evidence than smaller studies with limited scope or follow-up. Together, these priorities aim to advance more rigorous, species-specific, and policy-relevant research on the role of WASH in STH transmission and consequently address the questions most relevant to the achievement of STH control and elimination goals.

## Conclusions

This scoping review documented the way in which WASH factors have been examined in relation to STH transmission pathways, and how these factors have been conceptualised and measured. We identified a substantial and growing body of evidence examining the relationship between WASH factors, environmental exposures, and STH infection. There is a strong biological and practical rationale for the widely held consensus that WASH is integral to STH prevention. However, we identified several methodological and conceptual shortcomings in routinely conducted research, particularly in observational studies, as well as significant under-investigation of important aspects of the relationship between WASH and STH. These gaps and methodological issues limit the ability to develop consensus across studies and draw conclusions for programme design and public health practice. Rather than attempting to re-examine if WASH, whether as a comprehensive package of interventions or a specific single intervention is important for STH prevention, future research should address the evidence gaps identified in this review and incorporate the suggested methodological improvements to ensure that studies generate meaningful, comparable and generalisable evidence on how WASH conditions, access, and use influence STH transmission and control

## Supporting information

S1 Appendix Text A: Search terms; Table A: Data items; Table B: Included observational studies; Table C: Number of observational studies reporting on WASH relationship with infection and/or intensity for each WASH parameter; Table D: Included intervention studies. (DOCX)

S1 Data (XLSX)

S1 Checklist PRISMA Checklist. (DOCX)
