# Supplementary material for: Water, sanitation, and hygiene in soil-transmitted helminth control: A scoping review of evidence gaps across transmission pathways
Source: PLOS Glob Public Health. 2026 Oct 22;6(10):e0006196. doi: 10.1371/journal.pgph.0006196 (PMC13690228; doi:10.1371/journal.pgph.0006196)
Supplement: Supplementary file 1 [file pgph.0006196.s001.docx]

**Supplementary materials**

Text A: Search terms

Table A: Data items

Table B: Included observational studies

Table C: Number of observational studies reporting on WASH relationship with infection and/or intensity for each WASH parameter

Table D: Included intervention studies

**Text A: Search terms**

Heading 1: (helminth* or STH or intestinal worm* or ascari* or Trichuri* or Hookworm* or Necator or Ancylostoma or roundworm* or whipworm* or strongyloides ).ti,ab.

AND

Heading 2: (disinfect* or sanita* or water treatment or hygiene or toilet* or sewage or borehole* or standpipe or rainwater or latrine* or pit or open defecation or urination or fecal sludge or faecal sludge or slurry or effluent or shower* or piped water or pipe or surface water or irrigation or water supply or chlorination or treated water or water filter or water filtration or handwashing or hand washing or hand-washing or shoe*).ti,ab. or disinfection/ or sanitation/ or water treatment/ or hygiene/

AND

Heading 3: (incidence or prevalen* or epidemi* or infect* or surveill* or improve* or presence*).ti,ab

**Table A: Data items**

Based on the processes set out in the transmission conceptual framework, the following variables were extracted from included articles.

| **Variable** | **Assumptions/simplifications** |
| --- | --- |
| **Improved water source** | - Assumed that studies would include definition of ‘improved’ and provide information to enable categorisation as improved vs unimproved - Assumed not relevant for SS and most HW transmission |
| **Water treatment** | - Assumed not relevant for SS and most HW transmission |
| **Water storage** | - Assumed not relevant for SS and most HW transmission |
| **Improved sanitation** | - Assumed that studies would include definition of ‘improved’ and provide information to enable categorisation as improved vs unimproved |
| **Open defecation** | - Assumed studies would assess levels of open defecation and refer to open defecation practice explicitly |
| **Animal presence** | - Did not specify/restrict to certain types of animals (pets, livestock) or location (in house, any contact) |
| **Other environmental risk factors** | - Included any factors related to WASH, but not climatic conditions or vegetation |
| **Hygiene access (handwashing facilities)** | - Assumed bathing facilities not relevant. |
| **Handwashing** | - Included both reported and observed handwashing behaviour |
| **Nail hygiene** | - Assumed not relevant for HW/SS transmission |
| **Food safety** | - Assumed not relevant for SS and most HW transmission |
| **Geophagy** | - Assumed not relevant for SS and most HW transmission |
| **Shoe wearing** | - Assumed not relevant for AL/TT transmission |

SS=*Strongyloides stercoralis*; HW=hookworm species; AL=*Ascaris lumbricoides*; TT=*Trichuris trichiura*

***Table B: Included observational studies***

|  |  |  |  |  |  | **Species** | | | |
| --- | --- | --- | --- | --- | --- | --- | --- | --- | --- |
| **Author** | **Year** | **Title** | **Country** | **Rural/urban** | **# participants** | **AL** | **TT** | **HW** | **SS** |
| Adenusi, A.(1) | 2024 | Community‑based prevalence, intensity and risk factors associated with soil‑transmitted helminthiases and intestinal schistosomiasis in Apojola, Ogun state, southwest Nigeria | Nigeria | Rural | 283 | X |  | X |  |
| Adewale, B. (2) | 2023 | Monitoring the Status of Soil-Transmitted Helminthiases in Non-Endemic Implementation Units: A Case Study of Borgu in Northcentral Nigeria | Nigeria | Rural | 410 | X |  | X |  |
| Aiemjoy, K.(3) | 2017 | Epidemiology of Soil-Transmitted Helminth and Intestinal Protozoan Infections in Preschool-Aged Children in the Amhara Region of Ethiopia | Ethiopia | Rural | 214 | X |  |  |  |
| Ajjampur, S. (4) | 2021 | Epidemiology of soil transmitted helminths and risk analysis of hookworm infections in the community: Results from the DeWorm3 Trial in southern India | India | Rural | 6,089 |  |  | X |  |
| Akinsanya, B.(5) | 2021 | An investigation on the epidemiology and risk factors associated with soil-transmitted helminth infections in Ijebu East Local Government Area, Ogun State, Nigeria | Nigeria | Rural | 123 | X |  |  |  |
| Al-Mekhlafi, M. S. H. (6) | 2007 | An unceasing problem: soil-transmitted helminthiases in rural Malaysian communities | Malaysia | Rural | 610 | X | X | X |  |
| Al-Mekhlafi, H. (7) | 2019 | Prevalence and risk factors of Strongyloides stercoralis infection among Orang Asli schoolchildren: new insights into the epidemiology, transmission and diagnosis of strongyloidiasis in Malaysia | Malaysia | Rural | 1142 |  |  |  | X |
| Al-Murisi, W. (8) | 2022 | Schistosoma mansoni and soil-transmitted helminths among schoolchildren in An-Nadirah District, Ibb Governorate, Yemen after a decade of preventive chemotherapy | Yemen | Unspecified | 417 | X |  |  |  |
| Amahmid, O. (9) | 2005 | Assessment of the health hazards associated with wastewater reuse: transmission of geohelminthic infections (Marrakech, Morocco) | Morocco | Peri-urban | 610 | X | X |  |  |
| Amare, Y (10) | 2024 | A cross‑sectional study on the prevalence and associated risk factors for intestinal parasitic infections among under‑five aged children in Dabat primary hospital, north Gondar, Ethiopia | Ethiopia | Rural | 384 | X | X | X | X |
| Anegagrie, M. (11) | 2021 | Environmental characteristics around the household and their association with hookworm infection in rural communities from Bahir Dar, Amhara Region, Ethiopia | Ethiopia | rural and slightly urban | 792 |  |  | X |  |
| Aribodor O.B. (12) | 2018 | Risk factors and sociodemographic determinants of intestinal helminthiasis among children in schools that implemented the homegrown school feeding program in Ekwulobia, Anambra State, Southeast Nigeria | Nigeria | rural | 848 | X |  |  |  |
| Armiyanti, Y. (13) | 2020 | Hookworm infection and the risk factors among plantation workers in Jember, Indonesia | Indonesia | Rural | 277 | X |  | X |  |
| Asaolu, S.O. (14) | 2002 | Effect of water supply and sanitation on the prevalence and intensity of Ascaris lumbricoides among pre-school-age children in Ajebandele and Ifewara, Osun State, Nigeria. | Nigeria | Peri-urban | 516 | X |  |  |  |
| Astuti, E.P.(15) | 2024 | Prevalence and risk factors of soil transmitted helminth infections among school-aged children in Garut, Indonesia: Insights from a six-year deworming intervention | Indonesia | Rural | 386 | X | X |  |  |
| Aung, M. (16) | 2018 | Prevalence and associated risk factors of Strongyloides stercoralis infection in Lower Myanmar | Myanmar | Rural | 703 |  |  |  | X |
| Avokpaho, E. (17) | 2021 | Factors associated with soil-transmitted helminths infection in Benin: Findings from the DeWorm3 study | Benin | Rural | 6139 | X |  |  |  |
| Aw J.H.Y. (18) | 2021 | Novel statistical approaches to identify risk factors for STH infection in Timor-Leste | Timor-Leste | rural | 464 | X |  | X |  |
| Awharitoma, A. O. (19) | 2020 | Geo-helminth infections among school children in Ovbiogie and environ, Ovia North East Local Government Area, Edo State, Nigeria | Nigeria | Rural | 372 | X | X | X |  |
| Babamale, O. A. (20) | 2016 | Hookworm infection: a severe health problem in peri-urban community of North central, Nigeria | Nigeria | Peri-urban | 471 |  |  | X |  |
| Baker, J. M. (21) | 2018 | The associations between water and sanitation and hookworm infection using cross-sectional data from Togo's national deworming program | Togo | Nationwide | 33,363 |  |  | X |  |
| Barreto, M. L. (22) | 2010 | Impact of a citywide sanitation program in Northeast Brazil on intestinal parasites infection in young children | Brazil | Urban | 1,920 | X | X |  |  |
| Bartlett, A. W. (23) | 2022 | Burden and factors associated with schistosomiasis and soil-transmitted helminth infections among school-age children in Huambo, Uige and Zaire provinces, Angola | Angola | rural>urban | 3649 | X | X | X |  |
| Basualdo, J. A. (24) | 2007 | Intestinal parasitoses and environmental factors in a rural population of Argentina, 2002–2003 | Argentina | Rural | 505 | X | X |  |  |
| Belay, S (25) | 2024 | Pattern and predictor of hookworm reinfection among schoolchildren in three districts of Amhara Region, northwest Ethiopia | Ethiopia | Rural/urban | 403 |  |  | X |  |
| Belo, S. (26) | 2005 | Prevalence, behavioural and social factors associated with Schistosoma intercalatum and geohelminth infections in Sao Tome and Principe | São Tomé and Príncipe | Urban | 130 | X | X |  |  |
| Benjamin-Chung, J., A. (27) | 2015 | The Interaction of Deworming, Improved Sanitation, and Household Flooring with Soil-Transmitted Helminth Infection in Rural Bangladesh | Bangladesh | Rural | 1,630 | X | X |  |  |
| Bishop, H. G. (28) | 2022 | Ascaris lumbricoides: the risk factors and effects on growth of schoolchildren within Samaru, Zaria, Nigeria | Nigeria | Rural | 203 | X |  |  |  |
| Brandon-Mong, G. J. (29) | 2017 | Soil-transmitted helminths in Malaysia landscape: an aborigines study | Malaysia | Rural | 235 | X | X | X |  |
| Calegar, D. A. (30) | 2021 | Socioenvironmental Factors Influencing Distribution and Intensity of Soil-Transmitted Helminthiasis in the Brazilian Amazon: Challenges for the 2030 Agenda | Brazil | Urban | 349 | X | X | X |  |
| Campbell, S. J. (31) | 2016 | Water, sanitation and hygiene related risk factors for soil-transmitted helminth and Giardia duodenalis infections in rural communities in Timor-Leste | Timor Leste | Rural | 2152 | X |  | X |  |
| Campbell, S. J. (32) | 2017 | Water, Sanitation and Hygiene (WASH) and environmental risk factors for soil-transmitted helminth intensity of infection in Timor-Leste, using real time PCR | Timor Leste | rural | 2152 | X |  | X |  |
| Carneiro, F. F. (33) | 2002 | The risk of Ascaris lumbricoides infection in children as an environmental health indicator to guide preventive activities in Caparao’ and Alto Caparao’, Brazil | Brazil | Rural | 760 | X |  |  |  |
| Chongsuvivatwong, V. (34) | 1996 | Predictors for the risk of hookworm infection: experience from endemic villages in southern Thailand | Thailand | Rural | 245 |  |  | X |  |
| Chongsuvivatwong, V. (34) | 1996 | Predictors for the risk of hookworm infection: experience from endemic villages in southern Thailand | Thailand | Rural | 456 |  |  | X |  |
| Corrales, L. F. (35) | 2006 | Association between intestinal parasitic infections and type of sanitation system in rural El Salvador | El Salvador | Rural | 127 | X | X | X |  |
| Cundill, B. (36) | 2011 | Rates and intensity of re-infection with human helminths after treatment and the influence of individual, household, and environmental factors in a Brazilian community | Brazil | Rural | 642 |  |  | X |  |
| Damtie, D. (37) | 2021 | Prevalence and Associated Risk Factors of Intestinal Parasitic Infections among Pregnant Women Attending Antenatal Care in Yifag Health Center, Northwest Ethiopia | Ethiopia | Rural | 277 | X |  | X |  |
| Echazu, A., D. (38) | 2015 | Effect of Poor Access to Water and Sanitation As Risk Factors for Soil-Transmitted Helminth Infection: Selectiveness by the Infective Route | Argentina | Rural/urban | 432 | X | X | X | X |
| Efon-Ekangouo, A (39) | 2024 | Persistent transmission of soil-transmitted helminths despite 16 years of uninterrupted Mebendazole- andivermectin-based preventive chemotherapy in the Lomie Health District (East Region, Cameroon): The emergency of complementary control strategies | Cameroon | Rural | 491 | X | X |  |  |
| Elfu Feleke, B. (40) | 2018 | Epidemiology of hookworm infection in the school-age children: A comparative cross-sectional study | Ethiopia | rural and urban | 2390 |  |  | X |  |
| Ensink, J. (41) | 2005 | High risk of hookworm infection among wastewater farmers in Pakistan | Pakistan | Rural | 1,704 |  |  | X |  |
| Fetene, Y.(42) | 2021 | Determinants of Helminthic Infections and Anemia among Schoolchildren in Bahir Dar Zuria District, Northwest Ethiopia | Ethiopia | Rural | 394 |  |  | X |  |
| Forrer, A. (43) | 2015 | Risk profiling of hookworm infection and intensity in southern Lao People's Democratic Republic using Bayesian models | Lao PDR | Rural | 3,371 |  |  | X |  |
| Forrer, A. (44) | 2016 | Ivermectin Treatment and Sanitation Effectively Reduce Strongyloides stercoralis Infection Risk in Rural Communities in Cambodia | Cambodia | Rural | 3697 |  |  |  | X |
| Forrer, A. (45) | 2018 | Strongyloides stercoralis and hookworm co-infection: spatial distribution and determinants in Preah Vihear Province, Cambodia | Cambodia | Rural | 2502 |  |  | X | X |
| Forrer, A. (46) | 2019 | Strongyloides stercoralis: Spatial distribution of a highly prevalent and ubiquitous soil-transmitted helminth in Cambodia | Cambodia | Rural | 7246 |  |  |  | X |
| Freeman, M. C. (47) | 2015 | Associations between school- and household-level water, sanitation and hygiene conditions and soil-transmitted helminth infection among Kenyan school children | Kenya |  | 4,931 | X | X | X |  |
| Gabrie, J. A. (48) | 2014 | School hygiene and deworming are key protective factors for reduced transmission of soil-transmitted helminths among schoolchildren in Honduras | Honduras | Rural | 320 | X | X | X |  |
| Galgamuwa, L. (49) | 2016 | Factors Associated with the Prevalence of Ascaris Lumbricoides Infection among Preschool Children in a Plantation Community, Kandy District, Sri Lanka | Sri Lanka | Rural | 254 | X |  |  |  |
| Garn, J. V. (50) | 2016 | Ascaris lumbricoides Infection Following School-Based Deworming in Western Kenya: Assessing the Role of Pupils' School and Home Water, Sanitation, and Hygiene Exposures | Kenya |  | 4404 | X |  |  |  |
| Geissler, P. W. (51) | 1998 | Geophagy as a risk factor for geohelminth infections: A longitudinal study of Kenyan primary schoolchildren | Kenya | Rural | 200 | X | X |  |  |
| Gildner, T. E. (52) | 2020 | Market integration and soil-transmitted helminth infection among the Shuar of Amazonian Ecuador | Ecuador | Rural | 620 | X | X |  |  |
| Glickman, L. T. (53) | 1999 | Nematode intestinal parasites of children in rural Guinea, Africa: Prevalence and relationship to geophagia | Guinea | Rural | 286 | X | X | X | X |
| Goncalves, A. Q. (54) | 2016 | Prevalence of intestinal parasites and risk factors forspecific and multiple helminth infections in a remote city of the Brazilian Amazon | Brazil | Urban | 594 | X | X | X |  |
| Greenland, K. (55) | 2015 | The epidemiology of soil-transmitted helminths in Bihar State, India | India |  | 1157 | X |  | X |  |
| Grimes, J. E. T. (56) | 2017 | Sanitation, hookworm, anemia, stunting, and wasting in primary school children in southern Ethiopia: Baseline results from a study in 30 schools | Ethiopia |  | 3729 |  |  | X |  |
| Gunawardena, K. (57) | 2004 | Socio-economic and behavioural factors affecting the prevalence of Ascaris infection in a low-country tea plantation in Sri Lanka | Sri Lanka | Rural | 176 | X |  |  |  |
| Gunawardena, K. (58) | 2005 | Effects of climatic, socioeconomic and behavioural factors on the transmission of hookworm (Necator americanus) on two low-country plantations in Sri Lanka | Sri Lanka | Rural | 477 |  |  | X |  |
| Gunawardena, K. (59) | 2011 | Soil-Transmitted Helminth Infections among Plantation Sector Schoolchildren in Sri Lanka: Prevalence after Ten Years of Preventive Chemotherapy | Sri Lanka | Rural | 1,890 | X | X | X |  |
| Habbari, K. (60) | 2000 | Geohelminthic infections associated with raw wastewater reuse for agricultural purposes in Beni-Mellal, Morocco | Morocco | Rural | 1,999 | X | X |  |  |
| Hailegebriel, T. (61) | 2017 | Prevalence of intestinal parasitic infections and associated risk factors among students at Dona Berber primary school, Bahir Dar, Ethiopia | Ethiopia | Urban | 359 |  |  | X |  |
| Hailu Amare, H. (62) | 2020 | Helminth infections among rural schoolchildren in Southern Ethiopia: A cross-sectional multilevel and zero-inflated regression model | Ethiopia | Rural | 850 | X | X |  |  |
| Hailu, G. G. (63) | 2021 | Assessment of the prevalence of intestinal parasitic infections and associated habit and culture-related risk factors among primary schoolchildren in Debre Berhan town, Northeast Ethiopia | Ethiopia | Urban | 645 | X |  |  |  |
| Hailu, T. (64) | 2018 | Multivariate analysis of factors associated with Schistosoma mansoni and hookworm infection among primary school children in rural Bahir Dar, Northwest Ethiopia | Ethiopia | Rural and urban | 409 |  |  | X |  |
| Hailu, T. (65) | 2019 | Prevalence and determinant factors of hookworm infection among school age children in Jawe district, NorthWest Ethiopia | Ethiopia | Rural and urban | 333 |  |  | X |  |
| Hailu, T. (66) | 2022 | Determinant factors of chronic Strongyloides stercoralis infection among schoolchildren in Amhara National Regional State, northwest Ethiopia | Ethiopia | Rural and urban | 844 |  |  |  | X |
| Hajare, S. T. (67) | 2022 | Prevalence of Ascaris lumbricoides infections among elementary school children and associated risk factors from Southern Ethiopia | Ethiopia | Rural | 391 | X |  |  |  |
| Hall, A. (68) | 1994 | Strongyloides stercoralis in an urban slum community in Bangladesh: factors independently associated with infection | Bangladesh | Urban | 880 |  |  |  | X |
| Halliday, K. E. (69) | 2019 | Community-level epidemiology of soil-transmitted helminths in the context of school-based deworming: baseline results of a cluster randomised trial on the coast of Kenya | Kenya | Rural | 19,684 |  | X | X |  |
| Halpenny, C. M. (70) | 2013 | Regional, Household and Individual Factors that Influence Soil Transmitted Helminth Reinfection Dynamics in Preschool Children from Rural Indigenous Panama | Panama | Rural | 356 | X |  | X |  |
| Harrington, H. (71) | 2015 | Prevalence of soil-transmitted helminths in remote villages in East Kwaio, Solomon Islands | Solomon Islands | Rural | 402 |  |  | X |  |
| Henry, F. J. (72) | 1988 | Reinfection with Ascaris lumbricoides after chemotherapy: a comparative study in three villages with varying sanitation | St Lucia | Rural | 219 | X |  |  |  |
| Hohmann, H. (73) | 2001 | Relationship of intestinal parasites to the environment and to behavioral factors in children in the Bolikhamxay province of Lao PDR | Lao PDR | Rural | 709 | X | X | X |  |
| Hughes, A (74) | 2023 | Epidemiology of soil-transmitted helminths using quantitative PCR and risk factors for hookworm and *Necator americanus* infection in school children in Dak Lak province, Vietnam | Vietnam | Rural | 7710 |  |  | X |  |
| Humphries, D. (75) | 2011 | Epidemiology of Hookworm Infection in Kintampo North Municipality, Ghana: Patterns of Malaria Coinfection, Anemia, and Albendazole Treatment Failure | Ghana | Rural | 126 |  |  | X |  |
| Huth, S. von (76) | 2019 | Prevalence and potential risk factors for gastrointestinal parasitic infections in children in urban Bissau, Guinea-Bissau | Guinea Bissau | Urban | 1274 |  |  | X |  |
| Ibrahim, T. (77) | 2018 | Epidemiology of soil-transmitted helminths and Schistosoma mansoni: a base-line survey among school children, Ejaji, Ethiopia | Ethiopia | Urban | 340 | X |  | X |  |
| Ivan, E. (78) | 2013 | Helminthic infections rates and malaria in HIV infected pregnant women on anti-retroviral therapy in Rwanda | Rwanda | Rural/ peri-urban | 980 | X | X | X |  |
| Jarquin, C. (79) | 2016 | Population Density, Poor Sanitation, and Enteric Infections in Nueva Santa Rosa, Guatemala | Guatemala | Rural (80%) | 701 | X |  |  |  |
| Jeza, V. T. (80) | 2022 | Schistosomiasis, soil transmitted helminthiasis, and malaria co-infections among women of reproductive age in rural communities of Kwale County, coastal Kenya | Kenya | Rural | 534 |  |  | X |  |
| Jin, H. (81) | 2021 | Prevalence and Risk Factors of Intestinal Helminthiasis in Remote Mountainous Villages of Northern Lao PDR: A Cross-Sectional Study | Lao PDR | Rural | 161 |  |  | X |  |
| Jiraanankul, V. (82) | 2011 | Incidence and Risk Factors of Hookworm Infection in a Rural Community of Central Thailand | Thailand | Rural | 585 |  |  | X |  |
| Justine, N.C. (83) | 2024 | Prevalence, Infection Intensity, and Risk Factors for Soil transmitted Helminth Infections among School Children in Northwestern Tanzania | Tanzania | Rural | 728 | X | X |  |  |
| Kaliappan, S. P. (84) | 2013 | Prevalence and clustering of soil-transmitted helminth infections in a tribal area in southern India | India | Rural | 1237 | X |  | X |  |
| Kaliappan, S. (85) | 2022 | Soil-transmitted helminth infections after mass drug administration for lymphatic filariasis in rural southern India | India | Rural | 862 |  |  | X |  |
| Kepha, S. (86) | 2017 | Impact of single annual treatment and four-monthly treatment for hookworm and Ascaris lumbricoides, and factors associated with residual infection among Kenyan school children | Kenya |  | 1141 | X |  | X |  |
| Ketema, H. (87) | 2015 | Prevalence of Necator americanus infection and risk factors among school-age children in Mirab Abaya District, South Ethiopia | Ethiopia |  | 423 |  |  | X |  |
| Khieu, V. (88) | 2013 | Diagnosis, treatment and risk factors of Strongyloides stercoralis in schoolchildren in Cambodia | Cambodia | semi-rural | 461 |  |  |  | X |
| Khieu, V. (89) | 2014 | Strongyloides stercoralis infection and re-infection in a cohort of children in Cambodia | Cambodia | semi-rural | 302 |  |  |  | X |
| Khieu, V. (90) | 2014 | Prevalence and risk factors of Strongyloides stercoralis in Takeo Province, Cambodia | Cambodia | Rural | 2861 |  |  |  | X |
| Kim, S. H. (91) | 2020 | A first nation-wide assessment of soil-transmitted helminthiasis in Fijian primary schools, and factors associated with the infection, using a lymphatic filariasis transmission assessment survey as surveillance platform | Fiji |  | 1,839 | X |  | X |  |
| Kim, E.S. (92) | 2024 | Factors associated with hookworm and Schistosoma mansoni infections among school-aged children in Mayuge district, Uganda | Uganda | Rural | 1617 |  |  | X |  |
| Knopp, S. (93) | 2011 | From morbidity control to transmission control: time to change tactics against helminths on Unguja Island, Zanzibar | Tanzania - Zanzibar | Rural | 2,858 | X | X | X | X |
| Knopp, S.(93) | 2013 | From morbidity control to transmission control: time to change tactics against helminths on Unguja Island, Zanzibar. (Special Issue: CONTRAST alliance: optimized surveillance and sustainable control of schistosomiasis.) | Tanzania - Zanzibar | Rural | 1080 | X | X | X |  |
| Kounnavong, S. (94) | 2011 | Soil-transmitted helminth infections and risk factors in preschool children in southern rural Lao People’s Democratic Republic | Lao PDR | Rural | 570 | X | X | X |  |
| Krause, R. J. (95) | 2015 | Ascaris and hookworm transmission in preschool children from rural Panama: role of yard environment, soil eggs/larvae and hygiene and play behaviours | Panama | Rural | 167 HHs | X |  | X |  |
| Lakew, A. (96) | 2015 | Prevalence of intestinal parasites among street beggars in Jimma town, Southwest Ethiopia | Ethiopia | Urban | 116 |  |  | X |  |
| Lanker, K.C. (97) | 2023 | Prevalence and associated risk factors of intestinal parasitic infections among children in pastoralist and agro-pastoralist communities in the Adadle woreda of the Somali Regional State of Ethiopia | Ethiopia | Rural | 366 | X |  |  |  |
| Le, B. (98) | 2022 | Using quantitative PCR to identify opportunities to strengthen soil-transmitted helminth control in Solomon Islands: a cross-sectional epidemiological survey | Solomon islands | Rural | 830 |  | X | X |  |
| Leang, C (99) | 2025 | Geospatial distribution and risk factors of *Strongyloides stercoralis* in rural communities of Northeast Thailand | Thailand | Rural | 260 |  |  |  | X |
| Lee, V. J. (100) | 2007 | Hookworm infections in Singaporean soldiers after jungle training in Brunei Darussalam | Brunei | Rural | 113 |  |  | X |  |
| Loukouri, A. (101) | 2019 | Prevalence, Intensity of Soil-Transmitted Helminths, and Factors Associated with Infection: Importance in Control Program with Ivermectin and Albendazole in Eastern Côte d'Ivoire | Côte d’Ivoire | Rural | 1905 at baseline |  |  | X |  |
| Luoba, A. I. (102) | 2005 | Earth-eating and reinfection with intestinal helminths among pregnant and lactating women in western Kenya | Kenya | Rural | 825 | X | X | X |  |
| Mahmud, M. A. (103) | 2013 | Risk factors for intestinal parasitosis, anaemia, and malnutrition among school children in Ethiopia | Ethiopia | Urban/ rural | 600 |  |  | X |  |
| Masaku, J (104) | 2023 | Understanding factors responsible for the slow decline of soil-transmitted helminthiasis following seven rounds of annual mass drug administration (2012–2018) among school children in endemic counties of Kenya: A mixed method study | Kenya | Rural | 1874 | X | X | X |  |
| Matthys, B. (105) | 2007 | Risk factors for Schistosoma mansoni and hookworm in urban farming communities in western Côte d’Ivoire | Côte d’Ivoire | Urban |  |  |  | X |  |
| Mihrshahi, S. (106) | 2009 | The effectiveness of 4 monthly albendazole treatment in the reduction of soil-transmitted helminth infections in women of reproductive age in Vietnam | Vietnam | Rural | 366 | X | X | X |  |
| Misikir, S. W. (107) | 2020 | Prevalence of hookworm infection and associated factors among pregnant women attending antenatal care at governmental health centers in DEMBECHA district, north West Ethiopia, 2018 | Ethiopia | Urban and rural | 306 |  |  | X |  |
| Mohd Hanapi, I.R. (108) | 2024 | Intestinal parasitic infections and risk analysis among urban refugees in the Klang Valley, Malaysia | Malaysia | Rural | 418 | X | X | X |  |
| Moraes, L. R. S. (109) | 2004 | Impact of drainage and sewerage on intestinal nematode infections in poor urban areas in Salvador, Brazil | Brazil | Urban | 1,893 | X | X | X |  |
| Morales, M. L. (110) | 2019 | Strongyloides stercoralis Infection at Different Altitudes of the Cusco Region in Peru | Peru | Rural | 462 |  |  |  | X |
| Morales-Espinoza, E. M. (111) | 2003 | Intestinal parasites in children, in highly deprived areas of the border region of Chiapas, Mexico | Mexico | Rural | 1,148 | X | X |  |  |
| Muadica, A. S. (112) | 2021 | Risk associations for intestinal parasites in symptomatic and asymptomatic schoolchildren in central Mozambique | Mozambique |  | 1093 |  |  |  | X |
| Mubarak, M. Y. (113) | 2017 | Hygienic Behaviors and Risks for Ascariasis among College Students in Kabul, Afghanistan | Afghanistan | Urban | 520 | X |  |  |  |
| Mukutmoni, M (114) | 2023 | Perceptions and practices of urban slum-dwelling women concerning soil-transmitted helminths infections in Bangladesh: A cross-sectional study. | Bangladesh | Urban | 206 | X | X |  |  |
| Muñoz-Antoli, C., A. (115) | 2014 | Prevalence and risk factors related to intestinal parasites among children in Department of Rio San Juan, Nicaragua | Nicaragua | Rural and urban | 382 | X | X | X |  |
| Narain, K. (116) | 2000 | Prevalence of Trichuris trichiura in relation to socio-economic and behavioral determinants of exposure to infection in rural Assam | India | Rural | 580 |  | X |  |  |
| Nasr, N. A. (117) | 2013 | Towards an effective control programme of soil-transmitted helminth infections among Orang Asli in rural Malaysia. Part 1: prevalence and associated key factors | Malaysia | Rural | 484 |  |  |  |  |
| Nasr, N. A. (118) | 2020 | A holistic approach is needed to control the perpetual burden of soil-transmitted helminth infections among indigenous schoolchildren in Malaysia | Malaysia | rural | 1142 | X | X | X |  |
| Ndokeji, S. (119) | 2016 | Prevalence and intensity of schistosoma mansoni and hookworm infections among pre-school and school-aged children in Ilemela district, north-western Tanzania | Tanzania | rural | 454 |  |  | X |  |
| Nguyen, P. H. (120) | 2006 | Intestinal helminth infections among reproductive age women in Vietnam: prevalence, co-infection and risk factors | Vietnam | Nationwide | 5127 | X | X | X |  |
| Nikolay, B. (121) | 2015 | Understanding Heterogeneity in the Impact of National Neglected Tropical Disease Control Programmes: Evidence from School-Based Deworming in Kenya | Kenya | both | 16,146 | X |  | X |  |
| Nisha, M. (122) | 2020 | Risk factors associated with soil transmitted helminth (STH) infection in two indigenous communities in Malaysia | Malaysia | rural | 190 | X |  |  |  |
| Nishiura, H. (123) | 2002 | Ascaris lumbricoides among children in rural communities in the Northern Area, Pakistan: prevalence, intensity, and associated socio-cultural and behavioral risk factors | Pakistan | Rural | 492 | X |  |  |  |
| Nnolim, C. (124) | 2020 | Intestinal helminth infection and malnutrition among public primary school children in Ibadan, Nigeria | Nigeria | Rural | 485 | X | X | X | X |
| Noradilah, S. A. (125) | 2022 | Role of seasonal variation on the prevalence and risk factors of trichuriasis among the aboriginal community in Malaysia | Malaysia | rural | 473 |  | X |  |  |
| Norhayati, M. (126) | 1998 | Some risk factors of Ascaris and Trichuris infection in Malaysian aborigine (Orang Asli) children | Malaysia | Rural | 205 | X | X |  |  |
| Nshimiyimana, L (127) | 2025 | Water and sanitation factors associated with schistosomiasis and soil transmitted helminthiasis persistence in Rwanda | Rwanda | Rural | 2499 | X | X | X |  |
| Ogochukwu, C. O. (128) | 2015 | A cross-sectional study of Ascaris lumbricoides infection in a rural community in Ebonyi state, Nigeria: prevalence and risk factors | Nigeria | Rural | 212 | X |  |  |  |
| Okoyo, C. (129) | 2020 | Prevalence, intensity and associated risk factors of soil-transmitted helminth and schistosome infections in Kenya: Impact assessment after five rounds of mass drug administration in Kenya | Kenya |  | 9,801 | X | X | X |  |
| Okoyo, C. (130) | 2021 | Statistical Regression Model of Water, Sanitation, and Hygiene; Treatment Coverage; and Environmental Influences on School-Level Soil-Transmitted Helminths and Schistosome Prevalence in Kenya: Secondary Analysis of the National Deworming Program Data | Kenya | urban and rural | ~21492 | X | X | X |  |
| Olsen, A. (131) | 2001 | A study of risk factors for intestinal helminth infections using epidemiological and anthropological approaches | Kenya | Rural | 333 | X | X | X |  |
| Ortiz Valencia, L. I. (132) | 2005 | Spatial ascariasis risk estimation using socioeconomic variables. | Brazil | Urban | 1,550 | X |  |  |  |
| Oswald, W. E. (133) | 2017 | Association of community sanitation usage with soil-transmitted helminth infections among school-aged children in Amhara Region, Ethiopia | Ethiopia | Rural | 9818 | X | X | X |  |
| Oswald, W. E. (134) | 2019 | Domains of transmission and association of community, school, and household sanitation with soil-transmitted helminth infections among children in coastal Kenya | Kenya | Rural | 4104 |  | X | X |  |
| Parajuli, R. P. (135) | 2009 | Behavioral and Nutritional Factors and Geohelminth Infection Among Two Ethnic Groups in the Terai Region, Nepal | Nepal | Rural | 95 | X | X | X |  |
| Pham-Duc, P. (136) | 2013 | Ascaris lumbricoides and Trichuris trichiura infections associated with wastewater and human excreta use in agriculture in Vietnam | Vietnam | Rural | 1,425 | X | X |  |  |
| Phillips, A (137) | 2022 | Association between water, sanitation, and hygiene access and the prevalence of soil-transmitted helminth and schistosome infections in Wolayita, Ethiopia | Ethiopia | Rural | 15133 | X | X | X |  |
| Phiri, B. B. W. (138) | 2016 | Analysing risk factors of co-occurrence of schistosomiasis haematobium and hookworm using bivariate regression models: Case study of Chikwawa, Malawi | Malawi | Rural | 1642 |  |  | X |  |
| Phonekeo, S (139) | 2023 | Intestinal helminth infections and associated risk factors among adults in the Lao People’s Democratic Republic | Lao PDR | Rural/ urban | 2800 |  |  | X |  |
| Phongluxa, K., V. (140) | 2013 | Helminth infection in southern Laos: high prevalence and low awareness | Lao PDR | Rural | 704 |  |  | X |  |
| Putu Indah, B. A. (141) | 2020 | Prevalence and risk factors of soil-transmitted helminth infection among farmers in Gelgel Village, Klungkung District, Bali, Indonesia | Indonesia | Rural | 162 | X | X | X |  |
| Quintero, K (142) | 2012 | Household social determinants of ascariasis and trichuriasis in North Central Venezuela | Venezuela | Rural and urban | 3,388 | X | X |  |  |
| Riess, H. (143) | 2013 | Hookworm infection and environmental factors in mbeya region, Tanzania: a cross-sectional, population-based study | Tanzania | rural | 6,375 |  |  | X |  |
| Rollemberg, C. V. (144) | 2015 | Predicting frequency distribution and influence of sociodemographic and behavioral risk factors of Schistosoma mansoni infection and analysis of co-infection with intestinal parasites | Brazil | rural | 500 | X | X | X |  |
| Saathoff, E. (145) | 2002 | Geophagy and its association with geohelminth infection in rural schoolchildren from northern KwaZulu-Natal, South Africa | South Africa | Rural | 1,161 | X | X | X |  |
| Santarem, V.A. (146) | 2023 | Seroprevalence and associated risk factors of strongyloidiasis in indigenous communities and healthcare professionals from Brazil | Brazil | Rural | 610 |  |  |  | X |
| Schmidlin, T. (147) | 2013 | Effects of hygiene and defecation behavior on helminths and intestinal protozoa infections in Taabo, Côte d'Ivoire | Côte d'Ivoire | rural | 1894 |  | X | X |  |
| Senephansiri, P. (148) | 2017 | Status and Risk Factors of Strongyloides stercoralis Infection in Rural Communities of Xayaburi Province, Lao PDR | Lao PDR | rural | 516 |  |  |  | X |
| Sherkhonov, T. (149) | 2013 | National intestinal helminth survey among schoolchildren in Tajikistan: Prevalences, risk factors and perceptions | Tajikistan | Nationwide | 1,642 | X | X | X |  |
| Shiferaw, M. B. (150) | 2015 | Helminthiasis: Hookworm Infection Remains a Public Health Problem in Dera District, South Gondar, Ethiopia | Ethiopia | rural and urban | 464 |  |  | X |  |
| Sitotaw, B. (151) | 2019 | Prevalence of intestinal parasitic infections and associated risk factors among Jawi primary school children, Jawi town, north-west Ethiopia | Ethiopia | rural and urban | 406 |  |  | X |  |
| Sitotaw, B. (152) | 2020 | Prevalence of Intestinal Parasitic Infections and Associated Risk Factors among the First-Cycle Primary Schoolchildren in Sasiga District, Southwest Ethiopia | Ethiopia | rural | 383 | X |  | X |  |
| Soares Magalhaes, R. J. (153) | 2011 | Geographical analysis of the role of water supply and sanitation in the risk of helminth infections of children in West Africa | Ghana, Mali, Burkina Faso |  | 18,812 |  |  | X |  |
| Srinivasan, P. (154) | 2016 | Household and personal factors are sources of heterogenity in intestinal parasite clearance among Mexican children 6-15 months of age supplemented with vitamin A and zinc | Mexico | peri-urban | 707 | X |  |  |  |
| Steinmann, P. (155) | 2010 | Rapid appraisal of human intestinal helminth infections among schoolchildren in Osh oblast, Kyrgyzstan | Kyrgyzstan |  | 1,262 | X |  |  |  |
| Tadege, B., Z. (156) | 2022 | Assessment of the nail contamination with soil-transmitted helminths in schoolchildren in Jimma Town, Ethiopia | Ethiopia | Urban | 600 | X | X |  |  |
| Tapisha, B (157) | 2025 | Assessment of burden and risk factors associated with Soil-transmitted helminth 1 infections among adolescent girls in Katete District of Zambia: A Cross-Sectional Study | Zambia | ? | 206 | X |  |  |  |
| Tefera, E. (158) | 2017 | Prevalence and intensity of soil transmitted helminths among school children of Mendera Elementary School, Jimma, Southwest Ethiopia | Ethiopia | urban | 715 | X | X | X |  |
| Trang, D. T. (159) | 2006 | Low risk for helminth infection in wastewater-fed rice cultivation in Vietnam | Vietnam | Rural | 1,139 | X | X | X |  |
| Trang, D. T. (160) | 2007 | Helminth infections among people using wastewater and human excreta in peri-urban agriculture and aquaculture in Hanoi, Vietnam | Vietnam | peri-urban | 807 | X | X | X |  |
| Traub, R. J. (161) | 2004 | The prevalence, intensities and risk factors associated with geohelminth infection in tea-growing communities of Assam, India | India | Rural | 328 | X | X |  |  |
| Ugbomoiko, U. S. (162) | 2009 | Socio-environmental factors and ascariasis infection among school-aged children in Ilobu, Osun State, Nigeria | Nigeria | Rural | 440 | X |  |  |  |
| Vaz Nery, S. (163) | 2019 | Risk factors for infection with soil-transmitted helminths during an integrated community level water, sanitation, and hygiene and deworming intervention in Timor-Leste | Timor Leste | rural | 2,333 | X |  | X |  |
| Walker, M. (164) | 2011 | Individual Predisposition, Household Clustering and Risk Factors for Human Infection with Ascaris lumbricoides: New Epidemiological Insights | Bangladesh | Urban | 2,929 | X |  |  |  |
| Werunga, D. K. (165) | 2020 | Prevalence and Intensity of Intestinal Helminth Infections in Preschool Pupils in Lugari Subcounty, Kakamega County, Kenya | Kenya | rural & periurban | 130 | X |  |  |  |
| Witek-McManus, S. (166) | 2021 | Epidemiology of soil-transmitted helminths following sustained implementation of routine preventive chemotherapy: demographics and baseline results of a cluster randomised trial in southern Malawi | Malawi | rural | 6,102 |  |  | X |  |
| Wördemann, M (167) | 2006 | Prevalence and risk factors of intestinal parasites in Cuban children | Cuba |  | 1,320 | X | X | X |  |
| Xiao, P. L. (168) | 2015 | Prevalence and risk factors of Ascaris lumbricoides (Linnaeus, 1758), Trichuris trichiura (Linnaeus, 1771) and HBV infections in Southwestern China: a community-based cross sectional study | China | rural | 438 | X | X |  |  |
| Yajima, A. (169) | 2009 | High latrine coverage is not reducing the prevalence of soil-transmitted helminthiasis in Hoa Binh province, Vietnam | Vietnam |  | 155 | X | X | X |  |
| Yang, D. (170) | 2018 | Prevalence and Risk Factors of Ascaris lumbricoides, Trichuris trichiura and Cryptosporidium Infections in Elementary School Children in Southwestern China: A School-Based Cross-Sectional Study | China | rural | 321 | X | X |  |  |
| Yori, P. P. (171) | 2006 | Seroepidemiology of strongyloidiasis in the Peruvian Amazon | Peru | Rural | 908 |  |  |  | X |
| Young, S. L. (172) | 2007 | Association of geophagia with Ascaris, Trichuris and hookworm transmission in Zanzibar, Tanzania | Tanzania - Zanzibar | Rural | 970 | X | X | X |  |

**Table C: Number of observational studies reporting on WASH relationship with infection and/or intensity for each WASH parameter**

|  | **Number of studies** | | | |
| --- | --- | --- | --- | --- |
|  | ***AL [n=109]*** | ***TT (n=62)*** | ***HW (n=97)*** | ***SS (n=18)*** |
| **Improved water source** | 61 | 38 | 47 | 8 |
| **Water treatment** | 14 | 5 | 8 | 1 |
| **Water storage** | 6 | 4 | 4 | 0 |
| **Improved sanitation** | 93 | 60 | 93 | 16 |
| **Open defecation** | 32 | 22 | 38 | 8 |
| **Using excreta/wastewater in agriculture** | 11 | 10 | 7 | 0 |
| **Animal presence** | 13 | 9 | 17 | 6 |
| **Other environmental risk factors** | 27 | 16 | 25 | 6 |
| **Hygiene access (handwashing facilities)** | 14 | 6 | 13 | 1* |
| **Handwashing** | 51 | 30 | 40 | 6 |
| **Nail hygiene [nail biting]** | 17 [2] | 6 | 10 | 1 |
| **Food safety** | 26 | 13 | 20 | 2 |
| **Geophagy** | 13 | 10 | 11 | 3 |
| **Shoe wearing** | 17** | 12** | 50 | 13 |

*** use of river or stream for personal hygiene

** variable applied to specific species in risk-factor analysis
 **Table D: Included intervention studies**

| **Author** | **Year** | **Title** | **Design/ duration and follow up** | **Country** | **# partici-pants (Int/Cont)** | **Deworming treatment** | **Aims** | **Intervention/ setting** | **Reduction in prevalence, infection intensity or re-infection** | **Author interpretation of results** | **AL** | **HW** | **TT** | **SS** |
| --- | --- | --- | --- | --- | --- | --- | --- | --- | --- | --- | --- | --- | --- | --- |
| Al-Delaimy, AK et al (173) | 2014 | Developing and evaluating health education learning package (HELP) to control soil-transmitted helminth infections among Orang Asli children in Malaysia | Open label controlled intervention trial  6 months/ 6 months | Malaysia | 317 | Prior to intervention | Develop a health education learning package (HELP) about STH infections, and evaluate what impact such a package could have in terms of reducing the incidence and intensity of STH infections | Health education learning package (HELP): half day workshop for teachers, a teacher's guidebook to STH infections, posters, a comic book, a music video, a puppet show, drawing activities and an aid kit containing slippers, hand soap and nail clippers to help children put lessons into practice.  School-setting | The package significantly reduced the intensity of all three main STH infections and the prevalence of hookworm infections as a whole.  Although prevalence of A. lumbricoides and T. trichiura infections lower in intervention arm, the differences were not statistically significant. | Lack of significant prevalence reduction for A. lumbricoides and T. trichiura explained by easier mode of transmission compared with hookworm in heavily contaminated environment.  Low availability and usage of sanitation facilities in the study setting | X | X | X |  |
| Bieri (174) | 2013 | Health-Education Package to Prevent Worm Infections in Chinese Schoolchildren | Single-blind unmatched cluster RCT  One school year/ end of school year (10 months) | China | 1718 children  (825/893) |  | Prevention of helminth infections | Cartoon video plus discussion, poster, pamphlet, drawing and essay competitions.  School-setting | % children who washed their hands after using the toilet nearly twice as high in the intervention group  Incidence of STH infection was 50% lower in the intervention group | Helminth prevalence low at baseline. | X |  | X |  |
| Bird, C. et al (175) | 2014 | Do shoes reduce hookworm infection in school-aged children on Pemba Island, Zanzibar? A pragmatic trial | Non-blinded cluster RCT  One off distribution/ 6 months | Tanzania (Zanzibar) | 915 | At baseline | Determine if shoes reduce hookworm infection among SACs in Zanzibar | One time shoe distribution.  School-setting | No significant reduction | No difference in shoe wearing practice between control and intervention group. |  | X |  |  |
| Clarke, N. E. et al (176) | 2018 | (S)WASH-D for Worms: A pilot study investigating the differential impact of school- versus community-based integrated control programs for soil-transmitted helminths | A two-arm, non-randomized cluster intervention study  6 months/ 6 months | Timor Leste | 118/337 | At baseline and six months before follow-up (in one school before intervention completion). | 1. assess feasibility/ acceptability of conducting trial comparing school- vs community deworming and WASH 2. establish “proof of principle” that community-wide STH control is more effective than school-based approach | School-based WASH and deworming in 6 communities; 3 also received community-based programme  Schools and rural communities | Trend towards reduced odds of N. americanus infection among children who received the community-wide intervention | Study schools not randomized, discrepancies potentially biased results.  Household practices self-reported.  higher than expected baseline coverage of household latrines.  Remaining open defecation practice. |  | X |  |  |
| Clasen, T. et al (177) | 2014 | Effectiveness of a rural sanitation programme on diarrhoea, soil-transmitted helminth infection, and child malnutrition in Odisha, India: a cluster-randomised trial | Cluster RCT  12 months/ 18 months | India | 1919/ 1465/1916 | At baseline | assess eﬀectiveness of rural household sanitation intervention to prevent diarrhoea, STH, child malnutrition; Investigate eﬀect of intervention the Total Sanitation Campaign. | Latrine promotion and construction  Village-setting | No evidence that the intervention reduced prevalence or egg counts of all STH, or of A. lumbricoides, T. trichiura, or hookworm | Insufficient coverage and use/functionality of latrines.  No evidence that intervention reduced faecal exposure.  Short follow up relative to expected uptake of intervention and expected health outcomes. | X | X | X |  |
| Dumba (178) | 2013 | Design and implementation of participatory hygiene and sanitation transformation (PHAST) as a strategy to control soil-transmitted helminth infections in Luweero, Uganda | Randomised community intervention trial  Unspecified | Uganda | 357/370 | Every three months (unclear over what period). | To assess the effect of PHAST on intestinal helminth transmission in children under five years. | Sanitation, hygiene and health promotion + handwashing facility construction through Participatory Hygiene and Sanitation Transformation (PHAST).  Village-setting | There was an overall reduction in the prevalence of children infected with helminths after intervention. | Indication that the likelihood of children getting infected with helminths reduced in most of the experimented variables. | X | X | X |  |
| Ercumen, A. et al (179) | 2019 | Effects of water, sanitation, handwashing and nutritional interventions on soil-transmitted helminth infections in young children: A cluster-randomized controlled trial in rural Bangladesh | Cluster RCT  25 months/ 30 months | Bangladesh | 4169 women, 5657 children/  1382 women, 1530 children | 2/3 of children were dewormed within 6 months prior to assessment (2 months on average). | assess impact of individual and combined WASH and nutrition interventions on STH infections | Multiple arms: water treatment; sanitation; handwashing; combined water, sanitation, handwashing (WSH); nutrition + WSH.  Rural communities | Modest but sustained reductions in hookworm prevalence and intensity from water treatment and combined WASH interventions. | A larger difference in reinfection effect between arms may have been detected over a longer period. | X | X | X |  |
| Freeman, M. C. et al (180) | 2013 | The impact of a school-based hygiene, water quality and sanitation intervention on soil-transmitted helminth reinfection: a cluster-randomized trial | Cluster RCT  18 months/ follow-up 1 at 11 months, follow-up 2 at 21 months | Kenya | 354/293 | At baseline and follow up 1 and 2 (not during the intervention). | assess impact of school-based WASH program in reducing STH reinfection after MDA compared to MDA alone | Hygiene promotion, water treatment technology and sanitation infrastructure  School-setting | The intervention reduced reinfection prevalence and egg count of A. lumbricoides.  No evidence of significant intervention effects on overall prevalence and intensity of T. trichiura or hookworm. | Study did not cover non-school sources of infection.  Heterogeneity in intervention uptake.  Inaccurate diagnostics. | X | X | X |  |
| Gyorkos (181) | 2013 | Impact of Health Education on Soil-Transmitted Helminth Infections in Schoolchildren of the Peruvian Amazon: A Cluster-Randomized Controlled Trial | Cluster RCT  4 months of intervention and immediate follow up | Peru | 1,089 (518/571) | At baseline | to determine the effectiveness of a health hygiene education intervention on the occurrence of STH re-infection four months post-de-worming | Health, hygiene and sanitation promotion  School-setting | A. lumbricoides infection intensity statistically significantly lower (by 58%) in intervention schools.  No significant changes in hookworm spp. or T. trichiura intensity or “any STH” prevalence | Short intervention duration and follow up (four months overall)  Possibility of bias. | X | X | X |  |
| Hurlimann, E. et al (182) | 2018 | Effect of an integrated intervention package of preventive chemotherapy, community-led total sanitation and health education on the prevalence of helminth and intestinal protozoa infections in Côte d’Ivoire | village level randomized control trial  7 months/ 12 months | Côte d’Ivoire | 1482 | Annual treatment shortly after baseline survey, second round at eight months | assess effect of integrated intervention package of chemotherapy, community-led total sanitation (CLTS) and health education, on helminth and intestinal protozoa infections and knowledge, attitude, practice and beliefs on disease and WASH | Community-led total sanitation  Community-setting | No significant differences in STH reinfection rates between intervention and control communities | Imported infections from schools in non-open defecation free (ODF) communities and plantations not impacted by intervention.  Relatively low reinfection rates in control and intervention groups – too short follow-up after second deworming may have masked intervention effect. | X | X | X |  |
| Knee, J et al (183) | 2021 | Effects of an urban sanitation intervention on childhood enteric infection and diarrhea in Maputo, Mozambique: A controlled before-and-after trial | Controlled before and after study  13 months/ 24 months | Mozambique | 502/499 | At baseline | evaluate the impact of an onsite urban sanitation intervention on the prevalence of enteric infection, soil transmitted helminth reinfection, and diarrhea among children | Pour-flush toilets to septic tanks, installed for household clusters in urban settings  Urban compounds | No evidence that intervention affected the prevalence of any measured outcome  Some reduced prevalence of T. trichiura in children born into study sites after intervention relative to same age group at baseline | High faecal contamination and burden of infection means reductions in contamination and exposure may be insufficient to realize measurable health gains.  Intervention did not address all potential transmission pathways. | X |  | X |  |
| Makata, K. et al (184) | 2021 | Hand hygiene intervention to optimise soil-transmitted helminth infection control among primary school children: the Mikono Safi cluster randomised controlled trial in northwestern Tanzania | Cluster RCT  12 months/ 12 months after baseline | Tanzania | 1566/1515 | Prior to baseline survey and again at baseline for remaining infections. | Assess the effect of a combination intervention to enhance handwashing with soap on STH re-infxn following MDA | Hygiene promotion for children and parents, minimal environmental modification  School-setting | No effect on STH infection | Infection in the home environment.  Other transmission routes such as contaminated water or food.  Limited changes in school children’s handwashing behaviour. | X |  | X |  |
| Mahmud, MA et al (185) | 2015 | Efficacy of handwashing with soap and nail clipping on intestinal parasitic infections in school-aged children: a factorial cluster randomized controlled trial | Factorial Cluster RCT  6 months/ at end of 6 month intervention period | Ethiopia | 367  (Intervention arm: 91/93/94,87 in control) | Before screening to exclude children diagnosed positive | to assess the impact of handwashing with soap and nail clipping on the prevention of intestinal parasite reinfections | Promotion, demonstration and observation of handwashing with soap; regular soap provision; nail clipping by fieldworkers  Community-setting | 14% of children in handwashing with soap intervention group reinfected versus 29% in the control group.  17% of children in the nail clipping intervention group reinfected versus 26% in control. | The intensive follow-up and monitoring during this study made it such that the assessment of the observed intervention benefits was under rather ideal circumstances, and hence the study could possibly overestimate the effects when compared to usual conditions. | X | X |  |  |
| Nachaiwieng, R. et al (186) | 2024 | The impact of an integrated intervention program combining drug therapy with water, sanitation, and hygiene (WASH) education on reinfection with intestinal parasitic infections among the Karen hill tribe in northern Thailand | Quasi-experimental  6 months/ at 3 months and 6 months following implementation | Thailand | 350/200 | Prior to intervention and after follow up | Evaluate the effect of an integrated intervention program of drug therapy combined with WASH education on reinfection with IPIs among the Karen hill tribe in an endemic area of northern Thailand. | WASH education activities at school and community including assessment of hygiene practices, provision of towels, training in alcogel production.  School and community setting | Absolute reduction rates of T. trichiura infection were 50.0% and 80.8% at the 3-month and 6-month follow-up, respectively | Findings suggest that the intervention measures employed were effective in decreasing the prevalence of the parasitic infections over time. | X | X | X |  |
| Patil, S. R. et al (187) | 2014 | The effect of India's total sanitation campaign on defecation behaviors and child health in rural Madhya Pradesh: a cluster randomized controlled trial | Cluster RCT  21 months/ at end of intervention | India | 1683/ 1707. But only 1150 children tested for STH | None specified | measure effect of Total Sanitation Campaign on availability of individual household latrines, defecation behaviors, and child health (diarrhea, highly credible gastrointestinal illness [HCGI], parasitic infections, anemia, growth). | Total Sanitation Campaign: subsidies/promotion of improved HH latrines; school education, preschool toilets, community sanitation complexes, sanitation markets  Community-setting | The intervention did not improve child health measures | Relatively short follow-up period unlikely to show STH reduction  Poor implementation fidelity, coverage targets not met, evidence for ‘contamination’ in ¼ of control villages  Bias possible in self-reported outcomes for infections and open defecation behaviours. | X |  |  |  |
| Pickering, A. J. et al (188) | 2019 | Effects of single and integrated water, sanitation, handwashing, and nutrition interventions on child soil-transmitted helminth and Giardia infections: A cluster-randomized controlled trial in rural Kenya | Cluster RCT  24 months/ 27 months | Kenya | 9077 | National school-based MDA undertaken during study. 43% of participants dewormed in the past 6 months (deworming consumption similar across study arms). | assess the effects of WASH, handwashing, and nutrition interventions in various combinations on child STH infections 2 years post-intervention. | Multiple arms: water treatment; improved sanitation; handwashing with soap; combined water treatment sanitation and handwashing (WSH); combined WSH and nutrition  Rural communities | Results significant for A. lumbricoides only, in water treatment, WSH and WSH+N arms. | T. trichiura and hookworm were rarely detected, resulting in imprecise effect estimates. | X | X | X |  |
| Reese H. et al (189) | 2019 | Assessing longer-term effectiveness of a combined household-level piped water and sanitation intervention on childhood diarrhea, acute respiratory infection, soil-transmitted helminth infection, and nutritional status: a matched cohort study in rural Odisha, India | Matched cohort  36 months/ 5 years post implementation | India | 500 households | None specified | Assess the effectiveness of a community-level combined household piped water and sanitation intervention at least 5 years after intervention completion | Household toilets, bathing facilities and water connections  Community setting | Intervention was protective against child STH infection. | Substantial gains in WASH coverage, access, and use. |  | X |  |  |
| Sayasone, S et al (190) | 2024 | Integrated Eco-Health approach significantly reduces helminth infections in endemic Khong islands with emphasis on Schistosoma mekongi | Stepped-wedge trial  13 months/ 12 months after intervention | Lao PDR | 364 / 257 | Before and after intervention, then after follow up study | assess the impact of an Eco‑Health/One‑Health approach in combination with mass drug administration (MDA) to reduce these helminth infections | Latrine construction/ two health education campaigns  Community setting | Hookworm reduced by 17.9%  T. trichiura infections went down by 3.9%, from 6.2% to 2.3% on the intervention island by 3.6%, from 5.5% to 1.9% on the control island | The study findings suggest that the Eco‑Health/ One‑Health approach appears to be associated with a significant reduction in prevalence of S. mekongi and helminth co‑infections, particularly hookworm and T. trichiura  Latrine coverage increased significantly from ~40% in both intervention and control islands to 100% and 89% respectively |  | X | X |  |
| Steinmann, P. et al (191) | 2015 | Control of soil-transmitted helminthiasis in Yunnan province, People's Republic of China: experiences and lessons from a 5-year multi-intervention trial | Trial  36 months/ 24 months post implementation | China | 3121 | MDA at follow up | establish the feasibility and long-term community effectiveness of different STH control strategies in high endemicity settings. | Chemotherapy, health education and latrine construction  Community setting | Significant reduction in prevalence of T. trichiura and hookworm, with the highest reductions achieved when chemotherapy was combined with sanitation and health education. | Highest reductions in all species achieved when deworming was combined with sanitation and health education.  All households in the three intervention villages had access to running water. | X | X | X |  |
| Tadesse, G et al (192) | 2024 | Investigating the effect of a school-based WASH intervention on soil-transmitted helminth and schistosome infections and nutritional status of school children in Ethiopia: a quasi-experimental study | Quasi-experimental  22 months/ one year into delivery and at the end of the study | Ethiopia | 481 / 592 | At the start of the study and twice more annually | assessed the additional impact of improved WASH infrastructure and health education at schools on STH and SCH infections in Ethiopia | Provision of clean water by building water tanks to capture rainwater, installing piped water; latrine construction; health education  School setting | Reduction in the intensity of infection of hookworm and Ascaris lumbricoides compared to baseline in both arms | Sample size might be too small to detect a small impact, and impact might be masked by deworming.  Short follow up (2 years) might not pick up significant differences.  Relatively low prevalence of helminth infections may lead to small effect size of the intervention, if not null. 4. WASH and behaviour change interventions were focused on schools only; don't account for community exposure. | X | X |  |  |
| Vaz Nery, S. et al (193) | 2019 | WASH for WORMS: A Cluster-Randomized Controlled Trial of the Impact of a Community Integrated Water, Sanitation, and Hygiene and Deworming Intervention on Soil-Transmitted Helminth Infections | Cluster RCT  11 months/ 4 times over 24 months | Timor Leste | 1033 / 1108 | Every six months over two years (five rounds). | determine whether a community-based WASH programme had additional beneﬁts in reducing STH infections compared with community deworming alone | Improved water source provision, promotion of improved household sanitation and handwashing with soap  Rural communities | The integrated WASH and deworming intervention did not have an effect on infection with A. lumbricoides or N. americanus compared with deworming alone. | Over 1/3 of intervention arm continued open defecation; slippage in latrine coverage over time.  Improvement in WASH conditions in control arm potentially masked impact of WASH intervention.  Differences between arms may have been masked by deworming in all clusters. | X | X |  |  |
| Vonghachack Y et al (194) | 2023 | Improved latrines minimally impact Schistosoma mekongi transmission in Mekong islands | Experimental pre- and post-intervention assessment  12 months | Lao PDR | 462 / 412 | Before and after intervention | Investigate the impact of latrine implementation on helminth infection, placing particular emphasis on S. mekongi, O. viverrini, hookworm, and S. stercoralis. | Promotion of and subsidies for latrine construction  Community setting | Hookworm infection was significantly reduced by 22.6% from 48.8% to 26.2% (P<0.001)  S. stercoralis decreased by 12.0% from 43.1% to 31.1% - not statistically significant | Continued practice of open defecation, barefoot walking and other risk practices despite achieving 100% latrine coverage in intervention group. |  | X |  | X |
| Zeng, X. J. et al (195) | 2019 | Effect of integrated control intervention on soil-transmitted helminth infections in Jiangxi province in southeast China | Trial; retrospective analysis of cross sectional data  36 months/ 5 years | China |  | Annual MDA over the three years to the entire population except pregnant women and children under 3 | assess the status and trends of STH and report on integrated control interventions | health education, deworming, improved drinking water supply, sanitation provision, health and hygiene behaviour change  Community setting | Following control efforts with integrated control intervention strategies, STH prevalence in Jiangxi province experienced remarkable trend in decline between 1989 and 2014. |  | X | X | X |  |

References:

1.	Adenusi AA, Sheba KF, Ugwueze KT, Akinsola OJ, Adegbite RB, Nwanya VA, et al. Community-based prevalence, intensity and risk factors associated with soil-transmitted helminthiases and intestinal schistosomiasis in Apojola, Ogun state, southwest Nigeria. BMC Infect Dis. 2024;24(1):1302.

2.	Adewale B, Mogaji H, Balogun J, Balogun E, Olamiju F, Herbert D. Monitoring the Status of Soil-Transmitted Helminthiases in Non-Endemic Implementation Units: A Case Study of Borgu in Northcentral Nigeria. Pathogens. 2023;12(3):21.

3.	Aiemjoy K, Gebresillasie S, Stoller NE, Shiferaw A, Tadesse Z, Chanyalew M, et al. Epidemiology of Soil-Transmitted Helminth and Intestinal Protozoan Infections in Preschool-Aged Children in the Amhara Region of Ethiopia. Am J Trop Med Hyg. 2017;96(4):866-72.

4.	Ajjampur SSR, Kaliappan SP, Halliday KE, Palanisamy G, Farzana J, Manuel M, et al. Epidemiology of soil transmitted helminths and risk analysis of hookworm infections in the community: Results from the DeWorm3 Trial in southern India. PLoS Negl Trop Dis. 2021;15(4):e0009338.

5.	Akinsanya B, Taiwo A, Adedamola M, Okonofua C. An investigation on the epidemiology and risk factors associated with soil-transmitted helminth infections in Ijebu East Local Government Area, Ogun State, Nigeria. Scientific African. 2021;12(31).

6.	Al-Mekhlafi MS, Atiya AS, Lim YA, Mahdy AK, Ariffin WA, Abdullah HC, et al. An unceasing problem: soil-transmitted helminthiases in rural Malaysian communities. Southeast Asian J Trop Med Public Health. 2007;38(6):998-1007.

7.	Al-Mekhlafi HM, Nasr NA, Lim YAL, Elyana FN, Sady H, Atroosh WM, et al. Prevalence and risk factors of Strongyloides stercoralis infection among Orang Asli schoolchildren: new insights into the epidemiology, transmission and diagnosis of strongyloidiasis in Malaysia. Parasitology. 2019;146(12):1602-14.

8.	Al-Murisi WMS, Al-Mekhlafi AM, Mahdy MAK, Al-Haidari SA, Annuzaili DA, Thabit AAQ. Schistosoma mansoni and soil-transmitted helminths among schoolchildren in An-Nadirah District, Ibb Governorate, Yemen after a decade of preventive chemotherapy. PLoS ONE. 2022;17(8):e0273503.

9.	Amahmid O, Bouhoum K. Assessment of the health hazards associated with wastewater reuse: transmission of geohelminthic infections (Marrakech, Morocco). Int J Environ Health Res. 2005;15(2):127-33.

10.	Amare Y, Yohannes T, Tesfaye S. A cross-sectional study on the prevalence and associated risk factors for intestinal parasitic infections among under-five aged children in Dabat primary hospital, north Gondar, Ethiopia. J Parasit Dis. 2024;48(4):789-801.

11.	Anegagrie M, Lanfri S, Aramendia AA, Scavuzzo CM, Herrador Z, Benito A, et al. Environmental characteristics around the household and their association with hookworm infection in rural communities from Bahir Dar, Amhara Region, Ethiopia. PLoS Negl Trop Dis. 2021;15(6):e0009466.

12.	Aribodor OB, Ekwunife, C.A. Sam-Wobo, O.S., Aribodor, D.N. Risk Factors and Socio-Demographic Determinants of Intestinal Helminthiasis among Children in Schools that Implemented the Homegrown School Feeding Program in Ekwulobia, Anambra State, Southeast Nigeria. International Journal of Translational Medical Research and Public Health. 2018;2(1):1-10.

13.	Armiyanti Y, Utami WS, Nurdian Y, Ng JAS, Hermansyah B. Hookworm infection and the risk factors among plantation workers in Jember, Indonesia. Ann Trop Med Public Health. 2020;23(8):1324-9.

14.	Asaolu SO, Ofoezie IE, Odumuyiwa PA, Sowemimo OA, Ogunniyi TA. Effect of water supply and sanitation on the prevalence and intensity of Ascaris lumbricoides among pre-school-age children in Ajebandele and Ifewara, Osun State, Nigeria. Trans R Soc Trop Med Hyg. 2002;96(6):600-4.

15.	Astuti EP, Hendri J, Yuliasih Y, Sulaeman RP, Isnani T, Saputra S, et al. Prevalence and risk factors of soil transmitted helminth infections among school-aged children in Garut, Indonesia: insights from a six-year deworming intervention. Asian Pacific Journal of Tropical Medicine December. 2024;17(12):527-35.

16.	Aung MPPTHH, Hino A, Oo KM, Win KK, Maruyama H, Htike WW, et al. Prevalence and associated risk factors of Strongyloides stercoralis infection in Lower Myanmar. Trop Med Health. 2018;46(1).

17.	Avokpaho E, Houngbegnon P, Accrombessi M, Atindegla E, Yard E, Rubin Means A, et al. Factors associated with soil-transmitted helminths infection in Benin: Findings from the DeWorm3 study. PLoS Negl Trop Dis. 2021;15(8):e0009646.

18.	Aw Y, Clarke NE, Mayfield HJ, Lau CL, Richardson A, Nery SV. Novel statistical approaches to identify risk factors for soil-transmitted helminth infection in Timor-Leste. Int J Parasitol. 2021;51(9):729-39.

19.	Awharitoma AO, Idubor VO, Imalele EE. Geo-helminth infections among school children in Ovbiogie and environ, Ovia North East Local Government Area, Edo State, Nigeria. Niger J Parasitol. 2020;41(2):287-94.

20.	Babamale OA, Ugbomoiko US. Hookworm infection: a severe health problem in peri-urban community of North central, Nigeria. Trop. 2016;33(1):8-13.

21.	Baker JM, Trinies V, Bronzan RN, Dorkenoo AM, Garn JV, Sognikin S, et al. The associations between water and sanitation and hookworm infection using cross-sectional data from Togo's national deworming program. PLoS Negl Trop Dis. 2018;12(3):e0006374.

22.	Barreto ML, Genser B, Strina A, Teixeira MG, Assis AM, Rego RF, et al. Impact of a citywide sanitation program in Northeast Brazil on intestinal parasites infection in young children. Environ Health Perspect. 2010;118(11):1637-42.

23.	Bartlett AW, Sousa-Figueiredo JC, van Goor RC, Monaghan P, Lancaster W, Mugizi R, et al. Burden and factors associated with schistosomiasis and soil-transmitted helminth infections among school-age children in Huambo, Uige and Zaire provinces, Angola. Infect. 2022;11(1):73.

24.	Basualdo JA, Córdoba MA, de Luca MM, Ciarmela ML, Pezzani BC, Grenovero MS, et al. Intestinal parasitoses and environmental factors in a rural population of Argentina, 2002-2003. Rev Inst Med Trop Sao Paulo. 2007;49(4):251-5.

25.	Belay S, Alemu G, Hailu T. Pattern and predictor of hookworm re-infection among schoolchildren in three districts of Amhara Region, northwest Ethiopia. PLoS ONE. 2024;19(5):e0303502.

26.	Belo S, Rompão H, Gonçalves L, Grácio MA. Prevalence, behavioural and social factors associated with Schistosoma intercalatum and geohelminth infections in São Tomé and Principe. Parassitologia. 2005;47(2):227-31.

27.	Benjamin-Chung J, Nazneen A, Halder AK, Haque R, Siddique A, Uddin MS, et al. The Interaction of Deworming, Improved Sanitation, and Household Flooring with Soil-Transmitted Helminth Infection in Rural Bangladesh. PLoS Negl Trop Dis. 2015;9(12):e0004256.

28.	Bishop HG, Azeez Z, Ujah AO, Momoh SJ, Barwa J. Ascaris lumbricoides: the risk factors and effects on growth of schoolchildren within Samaru, Zaria, Nigeria. Science World Journal. 2022;17(2):286-90.

29.	Brandon-Mong GJ, Abdullah NA, Shukor N, Jaturas N, Richard RL, Choo JC, et al. Soil-transmitted helminths in Malaysia landscape: an aborigines study. Trop. 2017;34(2):363-74.

30.	Calegar DA, Bacelar PAA, Evangelista BBC, Monteiro KJL, Santos JPD, Almeida MM, et al. Socioenvironmental Factors Influencing Distribution and Intensity of Soil-Transmitted Helminthiasis in the Brazilian Amazon: Challenges for the 2030 Agenda. J Trop Med. 2021;2021.

31.	Campbell SJ, Nery SV, D'Este CA, Gray DJ, McCarthy JS, Traub RJ, et al. Water, sanitation and hygiene related risk factors for soil-transmitted helminth and Giardia duodenalis infections in rural communities in Timor-Leste. Int J Parasitol. 2016;46(12):771-9.

32.	Campbell SJ, Nery SV, Wardell R, D'Este CA, Gray DJ, McCarthy JS, et al. Water, Sanitation and Hygiene (WASH) and environmental risk factors for soil-transmitted helminth intensity of infection in Timor-Leste, using real time PCR. PLoS Negl Trop Dis. 2017;11(3):e0005393.

33.	Carneiro FF, Cifuentes E, Tellez-Rojo MM, Romieu I. The risk of Ascaris lumbricoides infection in children as an environmental health indicator to guide preventive activities in Caparaó and Alto Caparaó, Brazil. Bull World Health Organ. 2002;80(1):40-6.

34.	Chongsuvivatwong V, Pas-Ong S, McNeil D, Geater A, Duerawee M. Predictors for the risk of hookworm infection: experience from endemic villages in southern Thailand. Trans R Soc Trop Med Hyg. 1996;90(6):630-3.

35.	Corrales LF, Izurieta R, Moe CL. Association between intestinal parasitic infections and type of sanitation system in rural El Salvador. Trop Med Int Health. 2006;11(12):1821-31.

36.	Cundill B, Alexander N, Bethony JM, Diemert D, Pullan RL, Brooker S. Rates and intensity of re-infection with human helminths after treatment and the influence of individual, household, and environmental factors in a Brazilian community. Parasitology. 2011;138(11):1406-16.

37.	Damtie D, Liyih M. Prevalence and Associated Risk Factors of Intestinal Parasitic Infections among Pregnant Women Attending Antenatal Care in Yifag Health Center, Northwest Ethiopia. Can J Infect Dis Med Microbiol. 2021;2021.

38.	Echazu A, Bonanno D, Juarez M, Cajal SP, Heredia V, Caropresi S, et al. Effect of Poor Access to Water and Sanitation As Risk Factors for Soil-Transmitted Helminth Infection: Selectiveness by the Infective Route. PLoS Negl Trop Dis. 2015;9(9):e0004111.

39.	Efon-Ekangouo A, De-Gaulle VF, Emalio Y, Orish VN, Djune-Yemeli L, Simo-Simo ML, et al. Persistent transmission of soil-transmitted helminths despite 16 years of uninterrupted Mebendazole- and ivermectin-based preventive chemotherapy in the Lomie Health District (East Region, Cameroon): The emergency of complementary control strategies. PLoS Negl Trop Dis. 2024;18(9):e0012508.

40.	Elfu Feleke B. Epidemiology of hookworm infection in the school-age children: A comparative cross-sectional study. Iran J Parasitol. 2018;13(4):560-6.

41.	Ensink JH, van der Hoek W, Mukhtar M, Tahir Z, Amerasinghe FP. High risk of hookworm infection among wastewater farmers in Pakistan. Trans R Soc Trop Med Hyg. 2005;99(11):809-18.

42.	Fetene Y, Hailu T, Yimer M, Alemu M. Determinants of Helminthic Infections and Anemia among Schoolchildren in Bahir Dar Zuria District, Northwest Ethiopia. J Parasitol Res. 2021;2021.

43.	Forrer A, Vounatsou P, Sayasone S, Vonghachack Y, Bouakhasith D, Utzinger J, et al. Risk profiling of hookworm infection and intensity in southern Lao People's Democratic Republic using Bayesian models. PLoS Negl Trop Dis. 2015;9(3):e0003486.

44.	Forrer A, Khieu V, Schindler C, Schar F, Marti H, Char MC, et al. Ivermectin Treatment and Sanitation Effectively Reduce Strongyloides stercoralis Infection Risk in Rural Communities in Cambodia. PLoS Negl Trop Dis. 2016;10(8):e0004909.

45.	Forrer A, Khieu V, Schar F, Vounatsou P, Chammartin F, Marti H, et al. Strongyloides stercoralis and hookworm co-infection: spatial distribution and determinants in Preah Vihear Province, Cambodia. Parasit Vectors. 2018;11(1):33.

46.	Forrer A, Khieu V, Vounatsou P, Sithithaworn P, Ruantip S, Huy R, et al. Strongyloides stercoralis: Spatial distribution of a highly prevalent and ubiquitous soil-transmitted helminth in Cambodia. PLoS Negl Trop Dis. 2019;13(6):e0006943.

47.	Freeman MC, Chard AN, Nikolay B, Garn JV, Okoyo C, Kihara J, et al. Associations between school- and household-level water, sanitation and hygiene conditions and soil-transmitted helminth infection among Kenyan school children. Parasit Vectors. 2015;8:412.

48.	Gabrie JA, Rueda MM, Canales M, Gyorkos TW, Sanchez AL. School hygiene and deworming are key protective factors for reduced transmission of soil-transmitted helminths among schoolchildren in Honduras. Parasit Vectors. 2014;7:354.

49.	Galgamuwa L, Iddawela D, Dharmaratne SD. Factors Associated with the Prevalence of Ascaris Lumbricoides Infection among Preschool Children in a Plantation Community, Kandy District, Sri Lanka. Southeast Asian J Trop Med Public Health. 2016;47(6):1143-52.

50.	Garn JV, Mwandawiro CS, Nikolay B, Drews-Botsch CD, Kihara JH, Brooker SJ, et al. Ascaris lumbricoides Infection Following School-Based Deworming in Western Kenya: Assessing the Role of Pupils' School and Home Water, Sanitation, and Hygiene Exposures. Am J Trop Med Hyg. 2016;94(5):1045-54.

51.	Geissler PW, Mwaniki D, Thiong F, Friis H. Geophagy as a risk factor for geohelminth infections: a longitudinal study of Kenyan primary schoolchildren. Trans R Soc Trop Med Hyg. 1998;92(1):7-11.

52.	Gildner TE, Cepon-Robins TJ, Liebert MA, Urlacher SS, Schrock JM, Harrington CJ, et al. Market integration and soil-transmitted helminth infection among the Shuar of Amazonian Ecuador. PLoS ONE. 2020;15(7):e0236924.

53.	Glickman LT, Camara AO, Glickman NW, McCabe GP. Nematode intestinal parasites of children in rural Guinea, Africa: prevalence and relationship to geophagia. Int J Epidemiol. 1999;28(1):169-74.

54.	Goncalves AQ, Junqueira AC, Abellana R, Barrio PC, Terrazas WC, Sodre FC, et al. Prevalence of intestinal parasites and risk factors forspecific and multiple helminth infections in a remote city of the Brazilian Amazon. Rev Soc Bras Med Trop. 2016;49(1):119-24.

55.	Greenland K, Dixon R, Khan SA, Gunawardena K, Kihara JH, Smith JL, et al. The epidemiology of soil-transmitted helminths in Bihar State, India. PLoS Negl Trop Dis. 2015;9(5):e0003790.

56.	Grimes JET, Tadesse G, Gardiner IA, Yard E, Wuletaw Y, Templeton MR, et al. Sanitation, hookworm, anemia, stunting, and wasting in primary school children in southern Ethiopia: Baseline results from a study in 30 schools. PLoS Negl Trop Dis. 2017;11(10):e0005948.

57.	Gunawardena GS, Karunaweera ND, Ismail MM. Socio-economic and behavioural factors affecting the prevalence of Ascaris infection in a low-country tea plantation in Sri Lanka. Ann Trop Med Parasitol. 2004;98(6):615-21.

58.	Gunawardena GS, Karunaweera ND, Ismail MM. Effects of climatic, socio-economic and behavioural factors on the transmission of hookworm (Necator americanus) on two low-country plantations in Sri Lanka. Ann Trop Med Parasitol. 2005;99(6):601-9.

59.	Gunawardena K, Kumarendran B, Ebenezer R, Gunasingha MS, Pathmeswaran A, de Silva N. Soil-transmitted helminth infections among plantation sector schoolchildren in Sri Lanka: prevalence after ten years of preventive chemotherapy. PLoS Negl Trop Dis. 2011;5(9):e1341.

60.	Habbari K, Tifnouti A, Bitton G, Mandil A. Geohelminthic infections associated with raw wastewater reuse for agricultural purposes in Beni-Mellal, Morocco. Parasitol Int. 2000;48(3):249-54.

61.	Hailegebriel T. Prevalence of intestinal parasitic infections and associated risk factors among students at Dona Berber primary school, Bahir Dar, Ethiopia. BMC Infect Dis. 2017;17(1):362.

62.	Hailu Amare H, Lindtjorn B. Helminth infections among rural schoolchildren in Southern Ethiopia: A cross-sectional multilevel and zero-inflated regression model. PLoS Negl Trop Dis. 2020;14(12):e0008002.

63.	Hailu GG, Ayele ET. Assessment of the prevalence of intestinal parasitic infections and associated habit and culture-related risk factors among primary schoolchildren in Debre Berhan town, Northeast Ethiopia. BMC Public Health. 2021;21(1):112.

64.	Hailu T, Alemu M, Abera B, Mulu W, Yizengaw E, Genanew A, et al. Multivariate analysis of factors associated with Schistosoma mansoni and hookworm infection among primary school children in rural Bahir Dar, Northwest Ethiopia. Trop Dis Travel Med Vaccines. 2018;4(1).

65.	Hailu T, Mulu W, Abera B. Prevalence and determinant factors of hookworm infection among school age children in Jawe district, NorthWest Ethiopia. Afr Health Sci. 2019;19(3):2439-45.

66.	Hailu T, Nibret E, Amor A, Munshea A, Anegagrie M. Determinant factors of chronic Strongyloides stercoralis infection among schoolchildren in Amhara National Regional State, northwest Ethiopia. Acta Trop. 2022;226:106280.

67.	Hajare ST, Mulu T, Upadhye VJ, Chauhan NM, Eriso F. Prevalence of Ascaris lumbricoides infections among elementary school children and associated risk factors from Southern Ethiopia. J Parasitic Dis. 2022.

68.	Hall A, Conway DJ, Anwar KS, Rahman ML. Strongyloides stercoralis in an urban slum community in Bangladesh: factors independently associated with infection. Trans R Soc Trop Med Hyg. 1994;88(5):527-30.

69.	Halliday KE, Oswald WE, McHaro C, Beaumont E, Gichuki PM, Kepha S, et al. Community-level epidemiology of soil-transmitted helminths in the context of school-based deworming: baseline results of a cluster randomised trial on the coast of Kenya. PLoS Neglected Tropical Diseases. 2019;13(8).

70.	Halpenny CM, Paller C, Koski KG, Valdes VE, Scott ME. Regional, household and individual factors that influence soil transmitted helminth reinfection dynamics in preschool children from rural indigenous Panama. PLoS Negl Trop Dis. 2013;7(2):e2070.

71.	Harrington H, Bradbury R, Taeka J, Asugeni J, Asugeni V, Igeni T, et al. Prevalence of soil-transmitted helminths in remote villages in East Kwaio, Solomon Islands. Western pac. 2015;6(3):51-8.

72.	Henry FJ. Reinfection with Ascaris lumbricoides after chemotherapy: a comparative study in three villages with varying sanitation. Trans R Soc Trop Med Hyg. 1988;82(3):460-4.

73.	Hohmann H, Panzer S, Phimpachan C, Southivong C, Schelp FP. Relationship of intestinal parasites to the environment and to behavioral factors in children in the Bolikhamxay Province of Lao PDR. Southeast Asian J Trop Med Public Health. 2001;32(1):4-13.

74.	Hughes A, Ng-Nguyen D, Clarke NE, Dyer CEF, Hii SF, Clements ACA, et al. Epidemiology of soil-transmitted helminths using quantitative PCR and risk factors for hookworm and Necator americanus infection in school children in Dak Lak province, Vietnam. Parasit Vectors. 2023;16(1):213.

75.	Humphries D, Mosites E, Otchere J, Twum WA, Woo L, Jones-Sanpei H, et al. Epidemiology of hookworm infection in Kintampo North Municipality, Ghana: patterns of malaria coinfection, anemia, and albendazole treatment failure. Am J Trop Med Hyg. 2011;84(5):792-800.

76.	Huth Sv, Kofoed PE, Holmskov U. Prevalence and potential risk factors for gastrointestinal parasitic infections in children in urban Bissau, Guinea-Bissau. Transactions of the Royal Society of Tropical Medicine and Hygiene. 2019;113(9):545-54.

77.	Ibrahim T, Zemene E, Asres Y, Seyoum D, Tiruneh A, Gedefaw L, et al. Epidemiology of soil-transmitted helminths and Schistosoma mansoni: a base-line survey among school children, Ejaji, Ethiopia. J. 2018;12(12):1134-41.

78.	Ivan E, Crowther NJ, Mutimura E, Osuwat LO, Janssen S, Grobusch MP. Helminthic infections rates and malaria in HIV-infected pregnant women on anti-retroviral therapy in Rwanda. PLoS Negl Trop Dis. 2013;7(8):e2380.

79.	Jarquin C, Arnold BF, Munoz F, Lopez B, Cuellar VM, Thornton A, et al. Population Density, Poor Sanitation, and Enteric Infections in Nueva Santa Rosa, Guatemala. Am J Trop Med Hyg. 2016;94(4):912-9.

80.	Jeza VT, Mutuku F, Kaduka L, Mwandawiro C, Masaku J, Okoyo C, et al. Schistosomiasis, soil transmitted helminthiasis, and malaria co-infections among women of reproductive age in rural communities of Kwale County, coastal Kenya. BMC Public Health. 2022;22(1):136.

81.	Jin H, Ryu K, Lee D, Vonghachack Y, Choi MH, Hong ST, et al. Prevalence and Risk Factors of Intestinal Helminthiasis in Remote Mountainous Villages of Northern Lao PDR: A Cross-Sectional Study. Korean J Parasitol. 2021;59(2):131-8.

82.	Jiraanankul V, Aphijirawat W, Mungthin M, Khositnithikul R, Rangsin R, Traub RJ, et al. Incidence and risk factors of hookworm infection in a rural community of central Thailand. Am J Trop Med Hyg. 2011;84(4):594-8.

83.	Justine NC, Bhuko J, Rubagumya SL, Basinda NS, Ruganuza DM, Zinga MM, et al. Prevalence, Infection Intensity, and Risk Factors for Soil-transmitted Helminth Infections among School Children in Northwestern Tanzania. Pathogens. 2024;13(8):27.

84.	Kaliappan SP, George S, Francis MR, Kattula D, Sarkar R, Minz S, et al. Prevalence and clustering of soil-transmitted helminth infections in a tribal area in southern India. Trop Med Int Health. 2013;18(12):1452-62.

85.	Kaliappan S, Ramanujam K, Manuel M, Farzana J, Janagaraj V, Laxmanan S, et al. Soil-transmitted helminth infections after mass drug administration for lymphatic filariasis in rural southern India. Trop Med Int Health. 2022;27(1):81-91.

86.	Kepha S, Mwandawiro CS, Anderson RM, Pullan RL, Nuwaha F, Cano J, et al. Impact of single annual treatment and four-monthly treatment for hookworm and Ascaris lumbricoides, and factors associated with residual infection among Kenyan school children. Infect. 2017;6(1):30.

87.	Ketema H, Biruksew A, Mekonnen Z. Prevalence of Necator americanus infection and risk factors among school-age children in Mirab Abaya District, South Ethiopia. Asian Pac J Trop Dis. 2015;5(5):363-8.

88.	Khieu V, Schar F, Marti H, Sayasone S, Duong S, Muth S, et al. Diagnosis, treatment and risk factors of Strongyloides stercoralis in schoolchildren in Cambodia. PLoS Negl Trop Dis. 2013;7(2):e2035.

89.	Khieu V, Hattendorf J, Schar F, Marti H, Char MC, Muth S, et al. Strongyloides stercoralis infection and re-infection in a cohort of children in Cambodia. Parasitology International. 2014;63(5):708-12.

90.	Khieu V, Schar F, Marti H, Bless PJ, Char MC, Muth S, et al. Prevalence and risk factors of Strongyloides stercoralis in Takeo Province, Cambodia. Parasit Vectors. 2014;7:221.

91.	Kim SH, Stothard JR, Rinamalo M, Rainima-Qaniuci M, Talemaitoga N, Kama M, et al. A first nation-wide assessment of soil-transmitted helminthiasis in Fijian primary schools, and factors associated with the infection, using a lymphatic filariasis transmission assessment survey as surveillance platform. PLoS Negl Trop Dis. 2020;14(9):e0008511.

92.	Kim ES, Adriko M, Oseku KC, Lokure D, Webb EL, Sabapathy K. Factors associated with hookworm and Schistosoma mansoni infections among school-aged children in Mayuge district, Uganda. BMC Public Health. 2024;24(1):1620.

93.	Knopp S, Stothard JR, Rollinson D, Mohammed KA, Khamis IS, Marti H, et al. From morbidity control to transmission control: time to change tactics against helminths on Unguja Island, Zanzibar. Acta Trop. 2013;128(2):412-22.

94.	Kounnavong S, Vonglokham M, Houamboun K, Odermatt P, Boupha B. Soil-transmitted helminth infections and risk factors in preschool children in southern rural Lao People's Democratic Republic. Trans R Soc Trop Med Hyg. 2011;105(3):160-6.

95.	Krause RJ, Koski KG, Pons E, Sandoval N, Sinisterra O, Scott ME. Ascaris and hookworm transmission in preschool children from rural Panama: role of yard environment, soil eggs/larvae and hygiene and play behaviours. Parasitology. 2015;142(12):1543-54.

96.	Lakew A, Kibru G, Biruksew A. Prevalence of intestinal parasites among street beggars in Jimma town, Southwest Ethiopia. Asian Pac J Trop Dis. 2015;5(S1):S85-S8.

97.	Lanker KC, Muhummed AM, Cisse G, Zinsstag J, Hattendorf J, Yusuf RB, et al. Prevalence and associated risk factors of intestinal parasitic infections among children in pastoralist and agro-pastoralist communities in the Adadle woreda of the Somali Regional State of Ethiopia. PLoS Neglected Tropical Diseases. 2023;17(7 July) (no pagination).

98.	Le B, Clarke N, Hii SF, Byrne A, Zendejas-Heredia PA, Lake S, et al. Using quantitative PCR to identify opportunities to strengthen soil-transmitted helminth control in Solomon Islands: a cross-sectional epidemiological survey. PLoS Neglected Tropical Diseases. 2022;16(5).

99.	Leang C, Suwannatrai A, Suttiprapa S, Laha T, Sripa B. Geospatial distribution and risk factors of Strongyloides stercoralis in rural communities of Northeast Thailand. Trop Med Int Health. 2025;30(5):393-403.

100.	Lee VJ, Ong A, Lee NG, Lee WT, Fong KL, Lim PL. Hookworm infections in Singaporean soldiers after jungle training in Brunei Darussalam. Trans R Soc Trop Med Hyg. 2007;101(12):1214-8.

101.	Loukouri A, Méité A, Kouadio OK, Djè NN, Trayé-Bi G, Koudou BG, et al. Prevalence, Intensity of Soil-Transmitted Helminths, and Factors Associated with Infection: Importance in Control Program with Ivermectin and Albendazole in Eastern Côte d'Ivoire. J Trop Med. 2019;2019.

102.	Luoba AI, Wenzel Geissler P, Estambale B, Ouma JH, Alusala D, Ayah R, et al. Earth-eating and reinfection with intestinal helminths among pregnant and lactating women in western Kenya. Trop Med Int Health. 2005;10(3):220-7.

103.	Mahmud MA, Spigt M, Mulugeta Bezabih A, Lopez Pavon I, Dinant GJ, Blanco Velasco R. Risk factors for intestinal parasitosis, anaemia, and malnutrition among school children in Ethiopia. Pathog Glob Health. 2013;107(2):58-65.

104.	Masaku J, Okoyo C, Araka S, Musuva R, Njambi E, Njomo DW, et al. Understanding factors responsible for the slow decline of soil-transmitted helminthiasis following seven rounds of annual mass drug administration (2012-2018) among school children in endemic counties of Kenya: A mixed method study. PLoS Negl Trop Dis. 2023;17(5):e0011310.

105.	Matthys B, Tschannen AB, Tian-Bi NT, Comoé H, Diabaté S, Traoré M, et al. Risk factors for Schistosoma mansoni and hookworm in urban farming communities in western Côte d'Ivoire. Trop Med Int Health. 2007;12(6):709-23.

106.	Mihrshahi S, Casey GJ, Montresor A, Phuc TQ, Thach DT, Tien NT, et al. The effectiveness of 4 monthly albendazole treatment in the reduction of soil-transmitted helminth infections in women of reproductive age in Viet Nam. Int J Parasitol. 2009;39(9):1037-43.

107.	Misikir SW, Wobie M, Tariku MK, Bante SA. Prevalence of hookworm infection and associated factors among pregnant women attending antenatal care at governmental health centers in DEMBECHA district, north West Ethiopia, 2017. BMC Pregnancy Childbirth. 2020;20(1):457.

108.	Mohd Hanapi IR, Behnke JM, Sahimin N, Saifulazmi NF, Golam Mohammad Khan ASJ, Abdul Mutalib RNS, et al. Intestinal parasitic infections and risk analysis among urban refugees in the Klang Valley, Malaysia. Trans R Soc Trop Med Hyg. 2024;118(1):18-32.

109.	Moraes LR, Cancio JA, Cairncross S. Impact of drainage and sewerage on intestinal nematode infections in poor urban areas in Salvador, Brazil. Trans R Soc Trop Med Hyg. 2004;98(4):197-204.

110.	Morales ML, Lopez M, Ly P, Anjum S, Fernandez-Baca MV, Valdivia-Rodriguez AM, et al. Strongyloides stercoralis Infection at Different Altitudes of the Cusco Region in Peru. Am J Trop Med Hyg. 2019;101(2):422-7.

111.	Morales-Espinoza EM, Sánchez-Pérez HJ, García-Gil Mdel M, Vargas-Morales G, Méndez-Sánchez JD, Pérez-Ramírez M. Intestinal parasites in children, in highly deprived areas in the border region of Chiapas, Mexico. Salud Publica Mex. 2003;45(5):379-88.

112.	Muadica AS, Balasegaram S, Beebeejaun K, Koster PC, Bailo B, Hernandez-de-Mingo M, et al. Risk associations for intestinal parasites in symptomatic and asymptomatic schoolchildren in central Mozambique. Clin Microbiol Infect. 2021;27(4):624-9.

113.	Mubarak MY, Wagner AL, Carlson BF, Boulton ML. Hygienic Behaviors and Risks for Ascariasis among College Students in Kabul, Afghanistan. Am J Trop Med Hyg. 2017;97(2):563-6.

114.	Mukutmoni M, Liza FT, Parvin RA, Nath TC. Perceptions and practices of urban slum-dwelling women concerning soil-transmitted helminths infections in Bangladesh: A cross-sectional study. Parasite Epidemiol Control. 2023;21:e00291.

115.	Munoz-Antoli C, Pavon A, Marcilla A, Toledo R, Esteban JG. Prevalence and risk factors related to intestinal parasites among children in Department of Rio San Juan, Nicaragua. Trans R Soc Trop Med Hyg. 2014;108(12):774-82.

116.	Narain K, Rajguru SK, Mahanta J. Prevalence of Trichuris trichiura in relation to socio-economic & behavioural determinants of exposure to infection in rural Assam. Indian J Med Res. 2000;112:140-6.

117.	Nasr NA, Al-Mekhlafi HM, Ahmed A, Roslan MA, Bulgiba A. Towards an effective control programme of soil-transmitted helminth infections among Orang Asli in rural Malaysia. Part 1: prevalence and associated key factors. Parasit Vectors. 2013;6:27.

118.	Nasr NA, Al-Mekhlafi HM, Lim YAL, Elyana FN, Sady H, Atroosh WM, et al. A holistic approach is needed to control the perpetual burden of soil-transmitted helminth infections among indigenous schoolchildren in Malaysia. Pathog Glob Health. 2020;114(3):145-59.

119.	Ndokeji S, Mazigo HD, Temu M, Kishamawe C, Malenganisho W, Todd J, et al. Prevalence and intensity of schistosoma mansoni and hookworm infections among pre-school and school-aged children in Ilemela district, north-western Tanzania. Tanzan J Health Res. 2016;18(2).

120.	Nguyen PH, Nguyen KC, Nguyen TD, Le MB, Bern C, Flores R, et al. Intestinal helminth infections among reproductive age women in Vietnam: prevalence, co-infection and risk factors. Southeast Asian J Trop Med Public Health. 2006;37(5):865-74.

121.	Nikolay B, Mwandawiro CS, Kihara JH, Okoyo C, Cano J, Mwanje MT, et al. Understanding Heterogeneity in the Impact of National Neglected Tropical Disease Control Programmes: Evidence from School-Based Deworming in Kenya. PLoS Negl Trop Dis. 2015;9(9):e0004108.

122.	Nisha M, Aiman M, Asyhira N, Syafiq H, Atiqah N, Kumarasamy V, et al. Risk factors associated with soil transmitted helminth (STH) infection in two indigenous communities in Malaysia. Trop. 2020;37(2):379-88.

123.	Nishiura H, Imai H, Nakao H, Tsukino H, Changazi MA, Hussain GA, et al. Ascaris lumbricoides among children in rural communities in the Northern Area, Pakistan: prevalence, intensity, and associated socio-cultural and behavioral risk factors. Acta Trop. 2002;83(3):223-31.

124.	Nnolim C, Adekeye TA, Awobode HO. Intestinal helminth infection and malnutrition among public primary school children in Ibadan, Nigeria. Niger J Parasitol. 2020;41(1):49-55.

125.	Noradilah SA, Anuar TS, Lee IL. Role of seasonal variation on the prevalence and risk factors of trichuriasis among the aboriginal community in Malaysia. Helminthologia. 2022;59(1):55-63.

126.	Norhayati M, Oothuman P, Fatmah MS. Some risk factors of Ascaris and Trichuris infection in Malaysian aborigine (Orang Asli) children. Med J Malaysia. 1998;53(4):401-7.

127.	Nshimiyimana L, Mbituyumuremyi A, Ower A, Mbonigaba JB, Palacio K, Musarurwa C, et al. Water and sanitation factors associated with schistosomiasis and soil transmitted helminthiasis persistence in Rwanda. Sci. 2025;15(1):15415.

128.	Ogochukwu CO, Patience OU. A cross-sectional study of Ascaris lumbricoides infection in a rural community in Ebonyi state, Nigeria: prevalence and risk factors. Iranian Journal of Public Health. 2015;44(10):1430-2.

129.	Okoyo C, Campbell SJ, Williams K, Simiyu E, Owaga C, Mwandawiro C. Prevalence, intensity and associated risk factors of soil-transmitted helminth and schistosome infections in Kenya: Impact assessment after five rounds of mass drug administration in Kenya. PLoS Negl Trop Dis. 2020;14(10):e0008604.

130.	Okoyo C, Campbell SJ, Owaga C, Onyango N, Medley G, Mwandawiro C. Statistical Regression Model of Water, Sanitation, and Hygiene; Treatment Coverage; and Environmental Influences on School-Level Soil-Transmitted Helminths and Schistosome Prevalence in Kenya: Secondary Analysis of the National Deworming Program Data. Am J Trop Med Hyg. 2021;104(6):2251-63.

131.	Olsen A, Samuelsen H, Onyango-Ouma W. A study of risk factors for intestinal helminth infections using epidemiological and anthropological approaches. J Biosoc Sci. 2001;33(4):569-84.

132.	Valencia LI, Fortes Bde P, Medronho Rde A. Spatial ascariasis risk estimation using socioeconomic variables. Int J Environ Health Res. 2005;15(6):411-24.

133.	Oswald WE, Stewart AE, Kramer MR, Endeshaw T, Zerihun M, Melak B, et al. Association of community sanitation usage with soil-transmitted helminth infections among school-aged children in Amhara Region, Ethiopia. Parasit Vectors. 2017;10(1):91.

134.	Oswald WE, Halliday KE, McHaro C, Witek-McManus S, Kepha S, Gichuki PM, et al. Domains of transmission and association of community, school, and household sanitation with soil-transmitted helminth infections among children in coastal Kenya. PLoS Negl Trop Dis. 2019;13(11):e0007488.

135.	Parajuli RP, Umezaki M, Watanabe C. Behavioral and nutritional factors and geohelminth infection among two ethnic groups in the Terai region, Nepal. Am J Hum Biol. 2009;21(1):98-104.

136.	Pham-Duc P, Nguyen-Viet H, Hattendorf J, Zinsstag J, Phung-Dac C, Zurbrugg C, et al. Ascaris lumbricoides and Trichuris trichiura infections associated with wastewater and human excreta use in agriculture in Vietnam. Parasitology International. 2013;62(2):172-80.

137.	Phillips AE, Ower AK, Mekete K, Liyew EF, Maddren R, Belay H, et al. Association between water, sanitation, and hygiene access and the prevalence of soil-transmitted helminth and schistosome infections in Wolayita, Ethiopia. Parasit Vectors. 2022;15(1):410.

138.	Phiri BBW, Ngwira B, Kazembe LN. Analysing risk factors of co-occurrence of schistosomiasis haematobium and hookworm using bivariate regression models: Case study of Chikwawa, Malawi. Parasite Epidemiol Control. 2016;1(2):149-58.

139.	Phonekeo S, Kounnavong S, Vonglokham M, Siengsounthone L, Homsana A, Gummin S, et al. Intestinal helminth infections and associated risk factors among adults in the Lao People's Democratic Republic. Infect. 2023;12(1):61.

140.	Phongluxa K, Xayaseng V, Vonghachack Y, Akkhavong K, van Eeuwijk P, Odermatt P. Helminth infection in southern Laos: high prevalence and low awareness. Parasit Vectors. 2013;6(1):328.

141.	Putu Indah BA, Anak Agung GI, Heny A, Yoes Prijatna D. Prevalence and risk factors of soil-transmitted helminth infection among farmers in Gelgel Village, Klungkung District, Bali, Indonesia. Biodiversitas: Journal of Biological Diversity. 2020;21(4):1535-40.

142.	Quintero K, Durán C, Duri D, Medina F, Garcia J, Hidalgo G, et al. Household social determinants of ascariasis and trichuriasis in North Central Venezuela. Int Health. 2012;4(2):103-10.

143.	Riess H, Clowes P, Kroidl I, Kowuor DO, Nsojo A, Mangu C, et al. Hookworm infection and environmental factors in mbeya region, Tanzania: a cross-sectional, population-based study. PLoS Negl Trop Dis. 2013;7(9):e2408.

144.	Rollemberg CV, Silva MM, Rollemberg KC, Amorim FR, Lessa NM, Santos MD, et al. Predicting frequency distribution and influence of sociodemographic and behavioral risk factors of Schistosoma mansoni infection and analysis of co-infection with intestinal parasites. Geospat Health. 2015;10(1):303.

145.	Saathoff E, Olsen A, Kvalsvig JD, Geissler PW. Geophagy and its association with geohelminth infection in rural schoolchildren from northern KwaZulu-Natal, South Africa. Trans R Soc Trop Med Hyg. 2002;96(5):485-90.

146.	Santarem VA, Doline FR, Dos Santos JHF, Ferreira IB, Gomes BB, Meisel DMC, et al. Seroprevalence and associated risk factors of strongyloidiasis in indigenous communities and healthcare professionals from Brazil. PLoS Negl Trop Dis. 2023;17(4):e0011283.

147.	Schmidlin T, Hurlimann E, Silue KD, Yapi RB, Houngbedji C, Kouadio BA, et al. Effects of hygiene and defecation behavior on helminths and intestinal protozoa infections in Taabo, Cote d'Ivoire. PLoS ONE. 2013;8(6):e65722.

148.	Senephansiri P, Laummaunwai P, Laymanivong S, Boonmar T. Status and Risk Factors of Strongyloides stercoralis Infection in Rural Communities of Xayaburi Province, Lao PDR. Korean J Parasitol. 2017;55(5):569-73.

149.	Sherkhonov T, Yap P, Mammadov S, Sayfuddin K, Martinez P, Amoss WP, et al. National intestinal helminth survey among schoolchildren in Tajikistan: prevalences, risk factors and perceptions. Acta Trop. 2013;126(2):93-8.

150.	Shiferaw MB, Mengistu AD. Helminthiasis: Hookworm Infection Remains a Public Health Problem in Dera District, South Gondar, Ethiopia. PLoS ONE. 2015;10(12):e0144588.

151.	Sitotaw B, Mekuriaw H, Damtie D. Prevalence of intestinal parasitic infections and associated risk factors among Jawi primary school children, Jawi town, north-west Ethiopia. BMC Infect Dis. 2019;19(1):341.

152.	Sitotaw B, Shiferaw W. Prevalence of Intestinal Parasitic Infections and Associated Risk Factors among the First-Cycle Primary Schoolchildren in Sasiga District, Southwest Ethiopia. J Parasitol Res. 2020;2020.

153.	Soares Magalhães RJ, Barnett AG, Clements AC. Geographical analysis of the role of water supply and sanitation in the risk of helminth infections of children in West Africa. Proc Natl Acad Sci U S A. 2011;108(50):20084-9.

154.	Srinivasan P, Lawa HR, Rosado JL, Al Mamun A, Khatun M, Santos JI, et al. Household and personal factors are sources of heterogenity in intestinal parasite clearance among Mexican children 6-15 months of age supplemented with vitamin A and zinc. Acta Trop. 2016;156:48-56.

155.	Steinmann P, Usubalieva J, Imanalieva C, Minbaeva G, Stefiuk K, Jeandron A, et al. Rapid appraisal of human intestinal helminth infections among schoolchildren in Osh oblast, Kyrgyzstan. Acta Trop. 2010;116(3):178-84.

156.	Tadege B, Mekonnen Z, Dana D, Tiruneh A, Sharew B, Dereje E, et al. Assessment of the nail contamination with soil-transmitted helminths in schoolchildren in Jimma Town, Ethiopia. PLoS ONE. 2022;17(6):e0268792.

157.	Tapisha B, Hamwata WP, Sinyange D, Halwindi H, Muula AS. Assessment of burden and risk factors associated with Soil-transmitted helminth infections among adolescent girls in Katete District of Zambia: A Cross-Sectional Study. medRxiv. 2025;23.

158.	Tefera E, Belay T, Mekonnen SK, Zeynudin A, Belachew T. Prevalence and intensity of soil transmitted helminths among school children of Mendera Elementary School, Jimma, Southwest Ethiopia. Pan Afr Med J. 2017;27:88.

159.	Trang do T, van der Hoek W, Cam PD, Vinh KT, Hoa NV, Dalsgaard A. Low risk for helminth infection in wastewater-fed rice cultivation in Vietnam. J Water Health. 2006;4(3):321-31.

160.	Do TT, Mølbak K, Phung DC, Dalsgaard A. Helminth infections among people using wastewater and human excreta in peri-urban agriculture and aquaculture in Hanoi, Vietnam. Trop Med Int Health. 2007;12 Suppl 2:82-90.

161.	Traub RJ, Robertson ID, Irwin P, Mencke N, Andrew Thompson RC. The prevalence, intensities and risk factors associated with geohelminth infection in tea-growing communities of Assam, India. Trop Med Int Health. 2004;9(6):688-701.

162.	Ugbomoiko US, Dalumo V, Ofoezie IE, Obiezue RN. Socio-environmental factors and ascariasis infection among school-aged children in Ilobu, Osun State, Nigeria. Trans R Soc Trop Med Hyg. 2009;103(3):223-8.

163.	Vaz Nery S, Clarke NE, Richardson A, Traub R, McCarthy JS, Gray DJ, et al. Risk factors for infection with soil-transmitted helminths during an integrated community level water, sanitation, and hygiene and deworming intervention in Timor-Leste. Int J Parasitol. 2019;49(5):389-96.

164.	Walker M, Hall A, Basáñez MG. Individual predisposition, household clustering and risk factors for human infection with Ascaris lumbricoides: new epidemiological insights. PLoS Negl Trop Dis. 2011;5(4):e1047.

165.	Werunga DK, Omukunda EN, Korir JC. Prevalence and Intensity of Intestinal Helminth Infections in Preschool Pupils in Lugari Subcounty, Kakamega County, Kenya. J Parasitol Res. 2020;2020.

166.	Witek-McManus S, Simwanza J, Chisambi AB, Kepha S, Kamwendo Z, Mbwinja A, et al. Epidemiology of soil-transmitted helminths following sustained implementation of routine preventive chemotherapy: Demographics and baseline results of a cluster randomised trial in southern Malawi. PLoS Negl Trop Dis. 2021;15(5):e0009292.

167.	Wördemann M, Polman K, Menocal Heredia LT, Diaz RJ, Madurga AM, Núñez Fernández FA, et al. Prevalence and risk factors of intestinal parasites in Cuban children. Trop Med Int Health. 2006;11(12):1813-20.

168.	Xiao PL, Zhou YB, Chen Y, Yang Y, Shi Y, Gao JC, et al. Prevalence and risk factors of Ascaris lumbricoides (Linnaeus, 1758), Trichuris trichiura (Linnaeus, 1771) and HBV infections in Southwestern China: a community-based cross sectional study. Parasit Vectors. 2015;8:661.

169.	Yajima A, Jouquet P, Do TD, Dang TC, Tran CD, Orange D, et al. High latrine coverage is not reducing the prevalence of soil-transmitted helminthiasis in Hoa Binh province, Vietnam. Trans R Soc Trop Med Hyg. 2009;103(3):237-41.

170.	Yang D, Yang Y, Wang Y, Yang Y, Dong S, Chen Y, et al. Prevalence and Risk Factors of Ascaris lumbricoides, Trichuris trichiura and Cryptosporidium Infections in Elementary School Children in Southwestern China: A School-Based Cross-Sectional Study. Int J Environ Res Public Health. 2018;15(9):22.

171.	Yori PP, Kosek M, Gilman RH, Cordova J, Bern C, Chavez CB, et al. Seroepidemiology of strongyloidiasis in the Peruvian Amazon. Am J Trop Med Hyg. 2006;74(1):97-102.

172.	Young SL, Goodman D, Farag TH, Ali SM, Khatib MR, Khalfan SS, et al. Geophagia is not associated with Trichuris or hookworm transmission in Zanzibar, Tanzania. Trans R Soc Trop Med Hyg. 2007;101(8):766-72.

173.	Al-Delaimy AK, Al-Mekhlafi HM, Lim YA, Nasr NA, Sady H, Atroosh WM, et al. Developing and evaluating health education learning package (HELP) to control soil-transmitted helminth infections among Orang Asli children in Malaysia. Parasit Vectors. 2014;7:416.

174.	Bieri FA, Gray DJ, Williams GM, Raso G, Li YS, Yuan L, et al. Health-education package to prevent worm infections in Chinese schoolchildren. N Engl J Med. 2013;368(17):1603-12.

175.	Bird C, Ame S, Albonico M, Bickle Q. Do shoes reduce hookworm infection in school-aged children on Pemba Island, Zanzibar? A pragmatic trial. Trans R Soc Trop Med Hyg. 2014;108(5):297-304.

176.	Clarke NE, Clements ACA, Amaral S, Richardson A, McCarthy JS, McGown J, et al. (S)WASH-D for Worms: A pilot study investigating the differential impact of school- versus community-based integrated control programs for soil-transmitted helminths. PLoS Negl Trop Dis. 2018;12(5):e0006389.

177.	Clasen T, Boisson S, Routray P, Torondel B, Bell M, Cumming O, et al. Effectiveness of a rural sanitation programme on diarrhoea, soil-transmitted helminth infection, and child malnutrition in Odisha, India: a cluster-randomised trial. Lancet Glob Health. 2014;2(11):e645-53.

178.	Dumba R, Kaddu JB, Wabwire-Mangen F. Design and implementation of participatory hygiene and sanitation transformation (PHAST) as a strategy to control soil-transmitted helminth infections in Luweero, Uganda. Afr Health Sci. 2013;13(2):512-7.

179.	Ercumen A, Benjamin-Chung J, Arnold BF, Lin A, Hubbard AE, Stewart C, et al. Effects of water, sanitation, handwashing and nutritional interventions on soil-transmitted helminth infections in young children: A cluster-randomized controlled trial in rural Bangladesh. PLoS Negl Trop Dis. 2019;13(5):e0007323.

180.	Freeman MC, Clasen T, Brooker SJ, Akoko DO, Rheingans R. The impact of a school-based hygiene, water quality and sanitation intervention on soil-transmitted helminth reinfection: a cluster-randomized trial. Am J Trop Med Hyg. 2013;89(5):875-83.

181.	Gyorkos TW, Maheu-Giroux M, Blouin B, Casapia M. Impact of health education on soil-transmitted helminth infections in schoolchildren of the Peruvian Amazon: a cluster-randomized controlled trial. PLoS Negl Trop Dis. 2013;7(9):e2397.

182.	Hurlimann E, Silue KD, Zouzou F, Ouattara M, Schmidlin T, Yapi RB, et al. Effect of an integrated intervention package of preventive chemotherapy, community-led total sanitation and health education on the prevalence of helminth and intestinal protozoa infections in Cote d'Ivoire. Parasit Vectors. 2018;11(1):115.

183.	Knee J, Sumner T, Adriano Z, Anderson C, Bush F, Capone D, et al. Effects of an urban sanitation intervention on childhood enteric infection and diarrhea in Maputo, Mozambique: A controlled before-and-after trial. elife. 2021;10(04):09.

184.	Makata K, Ensink J, Ayieko P, Hansen C, Sichalwe S, Mngara J, et al. Hand hygiene intervention to optimise soil-transmitted helminth infection control among primary school children: the Mikono Safi cluster randomised controlled trial in northwestern Tanzania. BMC Med. 2021;19(1):125.

185.	Mahmud MA, Spigt M, Bezabih AM, Pavon IL, Dinant GJ, Velasco RB. Efficacy of Handwashing with Soap and Nail Clipping on Intestinal Parasitic Infections in School-Aged Children: A Factorial Cluster Randomized Controlled Trial. PLoS Med. 2015;12(6):e1001837; discussion e.

186.	Nachaiwieng W, Sanit S, Kongta N, Saingamsook J, Duangmano S, Pornprasert S, et al. The impact of an integrated intervention program combining drug therapy with water, sanitation, and hygiene (WASH) education on reinfection with intestinal parasitic infections among the Karen hill tribe in northern Thailand. Parasit Vectors. 2024;17(1):544.

187.	Patil SR, Arnold BF, Salvatore AL, Briceno B, Ganguly S, Colford JM, Jr., et al. The effect of India's total sanitation campaign on defecation behaviors and child health in rural Madhya Pradesh: a cluster randomized controlled trial. PLoS Med. 2014;11(8):e1001709.

188.	Pickering AJ, Njenga SM, Steinbaum L, Swarthout J, Lin A, Arnold BF, et al. Effects of single and integrated water, sanitation, handwashing, and nutrition interventions on child soil-transmitted helminth and Giardia infections: A cluster-randomized controlled trial in rural Kenya. PLoS Med. 2019;16(6):e1002841.

189.	Reese H, Routray P, Torondel B, Sinharoy SS, Mishra S, Freeman MC, et al. Assessing longer-term effectiveness of a combined household-level piped water and sanitation intervention on child diarrhoea, acute respiratory infection, soil-transmitted helminth infection and nutritional status: a matched cohort study in rural Odisha, India. Int J Epidemiol. 2019;48(6):1757-67.

190.	Sayasone S, Vonghachack Y, Xia S, Lv S, Zhou XN, Odermatt P. Integrated Eco-Health approach significantly reduces helminth infections in endemic Khong islands with emphasis on Schistosoma mekongi. Infect. 2024;13(1):57.

191.	Steinmann P, Yap P, Utzinger J, Du Z, Jiang J, Chen R, et al. Control of soil-transmitted helminthiasis in Yunnan province, People's Republic of China: experiences and lessons from a 5-year multi-intervention trial. (Special Issue: Progress in research, control and elimination of helminth infections in Asia.). Acta Trop. 2015;141(Part B):271-80.

192.	Tadesse G, Wuletaw Y, Mekete K, Sime H, Yard E, Appleby L, et al. Investigating the effect of a school-based WASH intervention on soil-transmitted helminth and schistosome infections and nutritional status of school children in Ethiopia: a quasi-experimental study. Parasit Vectors. 2024;17(1):130.

193.	Vaz Nery S, Traub RJ, McCarthy JS, Clarke NE, Amaral S, Llewellyn S, et al. WASH for WORMS: A Cluster-Randomized Controlled Trial of the Impact of a Community Integrated Water, Sanitation, and Hygiene and Deworming Intervention on Soil-Transmitted Helminth Infections. Am J Trop Med Hyg. 2019;100(3):750-61.

194.	Vonghachack Y, Odermatt P, Utzinger J, Sayasone S. Improved latrines minimally impact Schistosoma mekongi transmission in Mekong islands. Sci One Health. 2023;2:100038.

195.	Zeng XJ, Jiang WS, Xie SY, Chen YD, Gu XN, Ge J, et al. Effect of integrated control intervention on soil-transmitted helminth infections in Jiangxi province in southeast China. Acta Trop. 2019;194:148-54.
